# The *Candida albicans ENO1* gene encodes a transglutaminase involved in growth, cell division, morphogenesis, and osmotic protection

**DOI:** 10.1074/jbc.M117.810440

**Published:** 2018-01-31

**Authors:** Elizabeth Reyna-Beltrán, María Iranzo, Karla Grisel Calderón-González, Ricardo Mondragón-Flores, María Luisa Labra-Barrios, Salvador Mormeneo, Juan Pedro Luna-Arias

**Affiliations:** From the Departamentos de ‡ Biología Celular and; ¶ Bioquímica, Centro de Investigación y de Estudios Avanzados del Instituto Politécnico Nacional (Cinvestav-IPN), C.P. 07360, Ciudad de México, México and; the § Department de Microbiologia i Ecologia, Facultad de Farmacia, Unidad de Microbiología, Universitat de València, 46100 Valencia, España

**Keywords:** autophagy, Candida albicans, cell division, cell wall, transglutaminase, enolase 1, yeast-to-mycelium transition

## Abstract

*Candida albicans* is an opportunistic fungus that is part of the normal microflora commonly found in the human digestive tract and the normal mucosa or skin of healthy individuals. However, in immunocompromised individuals, it becomes a serious health concern and a threat to their lives and is ranked as the leading fungal infection in humans worldwide. As existing treatments for this infection are non-specific or under threat of developing resistance, there is a dire necessity to find new targets for designing specific drugs to defeat this fungus. Some authors reported the presence of the transglutaminase activity in *Candida* and *Saccharomyces*, but its identity remains unknown. We report here the phenotypic effects produced by the inhibition of transglutaminase enzymatic activity with cystamine, including growth inhibition of yeast cells, induction of autophagy in response to damage caused by cystamine, alteration of the normal yeast division pattern, changes in cell wall, and inhibition of the yeast-to-mycelium transition. The latter phenomenon was also observed in the *C. albicans* ATCC 26555 strain. Growth inhibition by cystamine was also determined in other *Candida* strains, demonstrating the importance of transglutaminase in these species. Finally, we identified enolase 1 as the cell wall protein responsible for TGase activity. After studying the inhibition of enzymatic activities with anti-CaEno1 antibodies and through bioinformatics studies, we suggest that the enolase and transglutaminase catalytic sites are localized in different domains of the protein. The aforementioned data indicate that TGase/Eno1 is a putative target for designing new drugs to control *C. albicans* infection.

## Introduction

Fungal infections cause more than 1.3 million deaths worldwide, principally in individuals who have HIV infections or two or more pathological conditions (1, 2). Although there are more than 150 species of *Candida*, ∼20 species are known to cause human infections; *Candida albicans* is the most frequent causative agent of candidiasis and is the leading fungal infection (3, 4). This opportunistic fungus is a human commensal that can be isolated from normal mucosae or cutaneous microflora of healthy individuals (2). However, when patients receive prolonged treatments with antibiotics, chemotherapy, or immunosuppressive agents or are in surgical intensive care units, their condition can turn this usually commensal yeast into a pathogen implicated in life-threatening invasive candidiasis (3–6). Another serious concern that must be considered is the increasing number of cases reporting *Candida* resistance to antifungal drugs (4). Thus, *Candida* infection constitutes a clinical problem worldwide due to the difficulty of treating systemic candidiasis (7). There is a dire necessity to find new molecular targets for developing new drugs against this fungus.

*C. albicans* is characterized by a complex interplay with its host by the expression of fungal virulence factors that result in adherence, invasion, and cell damage (8), which constitute a set of molecular tools that have evolved to overcome the defensive lines of body. Fungal cell wall is the main structure in contact with the host and is essential for cell integrity. It protects cells against several environmental stress conditions, including dehydration, osmotic changes, heat, cold, immune system response, or attack by other microorganisms (9–12). Moreover, it has a role in adhesion to host cells through adhesins, as well as in cross-talk with hosts through the glycan code (9). The cell wall is mainly composed of proteins, glycans, and lower amounts of chitin (9–12). Cell wall proteins, which are generally heavily mannosylated via *O*- and *N*-linkages, function as cross-linking enzymes, structural elements, adhesins, and environmental stress sensors, and they protect fungal cells from environmental change (9–13). Two classes of covalently-bound fungal cell wall proteins (CWPs)^3^ have been identified, glycosylphosphatidylinositol (GPI)-dependent CWPs and Pir-CWPs (9–11), that can be extracted by treating cell walls with mild alkali (alkali-sensitive linkage, ASL). The *Saccharomyces cerevisiae* Cwp2 (ScCwp2) is a very small GPI wall protein containing a Pir repeat involved in linking ScCwp2 to β-1,3-glycan to increase wall integrity (10, 13). There are other proteins that lack homology to Pir proteins, designated alkali-sensitive linkage cell wall proteins (ASL-CWPs), that are covalently linked by mild alkali-sensitive chemical bonds to the cell walls of *C. albicans* and *S. cerevisiae* (9, 10, 13). In addition, other proteins are linked to CWPs through disulfide bonds (14).

Covalent linkages are established between most wall components to provide stability to the cell wall. Proteins of the Gas family have been described as the main cross-linkers of wall polymers (11). However, other proteins are involved in this function. Transglutaminases (TGases) are multifunctional enzymes involved in several post-translational modifications, including protein cross-linking, amine incorporation, and deamination. The best known TGase activity is cross-linking through a transamidation reaction between the side chains of Gln and Lys residues, resulting in the formation of *N*-ϵ-(γ-glutamyl)lysine amide bonds (15). These enzymes are usually Ca^2+^-dependent, although in the case of some microorganisms and rodent intestinal mucosa, TGases also act as Ca^2+^-independent enzymes (16, 17). TGases are widely distributed in animals, plants, and microorganisms and have key roles in several biological processes, including growth regulation, differentiation, cellular adhesion, and maintenance of tissue integrity. Human TGase 2 is involved in the stabilization of the extracellular matrix and modulates the fibronectin–integrin interaction (18, 19). It is involved in a number of diseases, including Alzheimer's disease, Huntington's disease, Crohn's disease, fibrosis, cancer metastasis, and other diseases (20). In addition, TGase is also involved in cross-linking cell wall components in *Chlamydomonas reinhardtii* (21). Given the importance of TGases in the development of serious diseases, much research has focused on exploring specific TGase inhibitors with a therapeutic purpose (22).

TGase activity was previously reported in the cell walls of *C. albicans* and *S. cerevisiae*. This enzyme establishes covalent cross-links between proteins, and its activity is affected by the specific inhibitor cystamine, which decreases the incorporation of several proteins into the cell wall and avoids regeneration of protoplasts, as well as the yeast-to-mycelium transition, indicating a role in the formation of covalent cross-links between wall proteins and chitin and/or glucan (23, 24). In this work, we report the identification of enolase 1 as the cell wall protein that has TGase activity, as well as the phenotypic changes produced by inhibition of TGase. Taken together, our data indicate that TGase is a putative target for designing new drugs to control *C. albicans* infection.

## Results

### Determination of transglutaminase activity in the cell walls of C. albicans

**Figure 1.**
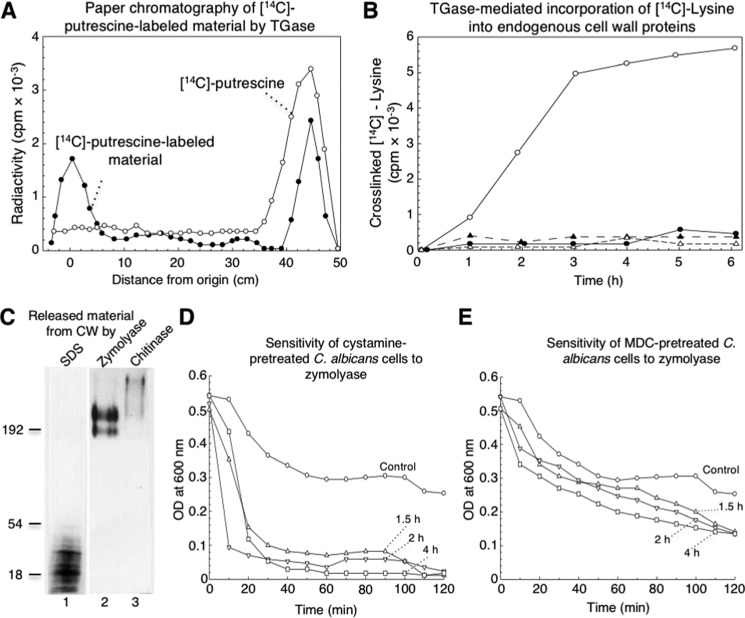
**[^14^C]Lysine cross-linked by TGase activity was found in low- and high-molecular-weight cell wall proteins and inhibition of TGase enzyme by cystamine and MDC induced changes in cell wall that sensitized cells to zymolyase treatment.** *A*, paper chromatography of [^14^C]putrescine-labeled material performed by *C. albicans* TGase activity and released from CW by zymolyase (*filled circles*). Solubilized fraction containing 20,000 cpm analyzed by paper chromatography was described under the “Experimental procedures.” [^14^C]Putrescine substrate was also analyzed as a control (*open circles*). *B*, transglutaminase-mediated incorporation of [^14^C]lysine into endogenous cell wall proteins. Reaction mixtures containing similar aliquots of cell walls and 2.5 μCi of [^14^C]lysine with no cystamine (*open circles*) or with 50 mm cystamine (*filled circles*), boiled enzymatic source (*open triangles*), or 2 mm EDTA (*filled triangles*) were incubated for the indicated times; radioactivity in TCA-precipitable material was quantified. *C*, labeled samples with [^14^C]lysine were sequentially released from cell walls by SDS, zymolyase, and chitinase and analyzed by 10% SDS-PAGE and fluorography. 10,000 cpm of labeled fractions were loaded in each lane. *Lane 1,* SDS-released material; *lane 2*, zymolyase-released material; *lane 3*, chitinase-released material. *D* and *E,* inhibition of TGase by cystamine and MDC increased sensitivity of *C. albicans* cells to treatment with zymolyase. Cells of *C. albicans* (adjusted to OD_600 nm_ = 0.5) previously incubated without (*circles*) or with 100 mm cystamine (*D*) or 3.5 mm MDC (*E*) for 1.5 h (*triangles*), 2 h (*inverted triangles*), and 4 h (*squares*) were treated with 50 μg ml^−1^ zymolyase 20T for up to 120 min. At the indicated times, OD_600 nm_ of cultures was monitored.

The standard protocol to determine TGase activity measures the incorporation of radioactive putrescine as a diamino acid analogue into TCA-precipitable material (23, 24). To determine TGase activity in cell walls (CW), we used a similar protocol, except that purified cell walls were used as the source of both TGase enzyme and endogenous acceptors. Using this procedure, there were many quantitative problems due to the non-specific adsorption of putrescine, which produced high levels of radioactivity in the negative controls. To confirm the presence of radioactive putrescine cross-linked to proteins in the TCA-precipitable material, this fraction was solubilized with zymolyase and analyzed by paper chromatography (Fig. 1*A*). Nearly 40% of total [^14^C]putrescine radioactivity was found at the origin, indicating that the substrate was cross-linked to high-molecular-weight molecules. Approximately 40% of radioactivity corresponded to unincorporated [^14^C]putrescine, whereas the remaining radioactivity was distributed through the paper. This was likely due to non-specific adsorption, as confirmed by paper chromatography performed with radioactive putrescine alone (Fig. 1*A*).

The use of either radioactive putrescine or lysine as substrates for TGase from guinea pig liver was evaluated (Table S1). In this assay, the incorporation of radioactive substrates was similar, as well as the inhibition of the TGase activity observed with cystamine. Therefore, we determined TGase activity using [^14^C]lysine and purified *C. albicans* cell walls. TGase activity was dependent on time up to 3–4 h (Fig. 1*B*). The presence of EDTA inhibited TGase activity, similar to that produced by cystamine or boiling preparations used in the assay (Fig. 1*B*).

### Characterization of TGase acceptors from cell walls

To study wall-endogenous acceptors of TGase, purified cell walls were incubated with [^14^C]lysine as described and sequentially extracted with SDS, zymolyase, and chitinase. In each case, solubilized materials were analyzed by 10% SDS-PAGE and fluorography. From the total amount of radioactivity incorporated into cell walls by the action of TGase activity, nearly 40% was solubilized by SDS, whereas zymolyase released 15% and chitinase 23%, respectively. SDS-solubilized proteins had apparent molecular masses of less than 50 kDa, whereas the material released by either zymolyase or chitinase showed molecular masses greater than 180 kDa, *i.e.* the chitinase-released proteins of the highest molecular mass (Fig. 1*C*). The mass spectrometry analysis of the proteins solubilized with 2% SDS in the radioactive region of the gel shown in Fig. 1*C* revealed the presence of 1046 unique proteins (Table S2). These proteins were classified with the Gene Ontology Panther Classification System (Fig. S1). The most abundant classes for molecular function corresponded to proteins with catalytic, binding, structural, and transporter activities. In the case of protein classes, the most abundant proteins were nucleic acid-binding, oxidoreductases, hydrolases, and transferases. In the case of the proteins released with zymolyase, we identified 37 proteins (Table S3). Panther classified these proteins according to molecular function in seven categories, with catalytic, binding, and structural the most frequent categories (Fig. S2). According to protein class, they were categorized in 10 groups. The classes with more proteins were nucleic acid-binding proteins, oxidoreductases, hydrolases, and transporters. Finally, the treatment with chitinase released 41 proteins (Table S4), which were classified by Panther in six molecular function categories, with catalytic, binding, structural, and transporter activities the most represented, although according to molecular class, they were grouped in nine categories. The two most represented were nucleic acid-binding and oxidoreductase proteins (Fig. S3). Remarkably, enolase 1 was found in the proteins sequentially extracted with 2% SDS, zymolyase, and chitinase.

### TGase activity is a key molecule that confers stability to the C. albicans cell wall

To study the role of TGases in the osmosensitivity of cells after digestion with zymolyase, *C. albicans* yeast cells were incubated with 100 mm cystamine or 3.5 mm MDC for 1.5, 2, and 4 h before treatment with glucanases for up to 120 min. Cell cultures treated with zymolyase alone showed a decrease in absorbance at 600 nm of 40% at 60 min that was maintained for the entire incubation time, while cells incubated with cystamine for different times achieved a loss in absorbance of ∼70% at 20 min of incubation with zymolyase, which slowly decreased up to 95% at 100 min (Fig. 1*D*). In the case of impaired osmotic protection by the inhibition of TGase activity by MDC, this effect was not as dramatic as that shown with cystamine. A decrease of 30% in absorbance was observed in the first 20 min, reaching a maximum of 70% at 120 min of incubation (Fig. 1*E*). Thus, the inhibition of TGase activity by cystamine and MDC affects the osmotic protection of the cell wall after glucan digestion with zymolyase.

**Figure 2.**
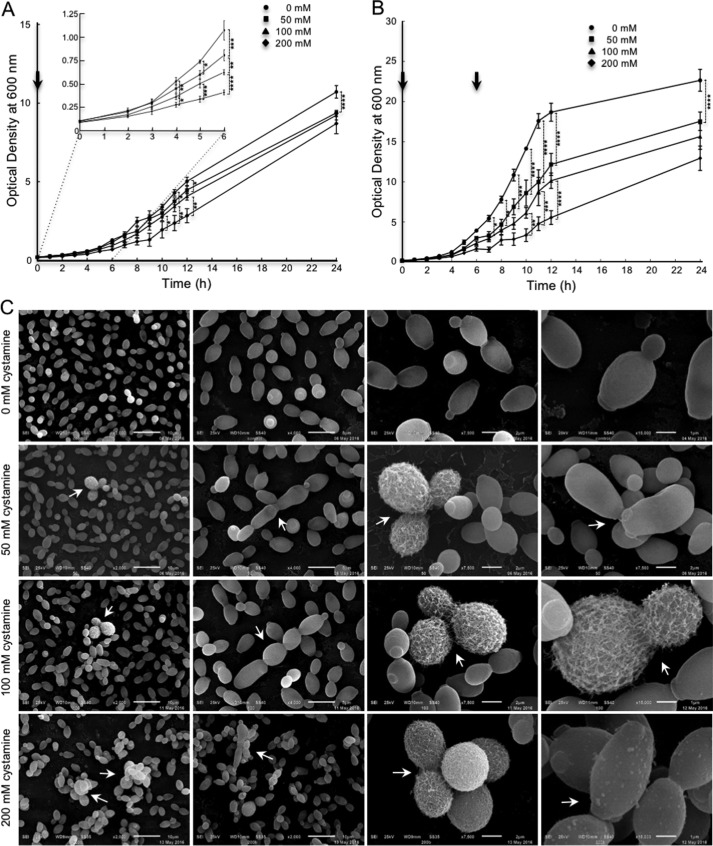
**Effect of inhibition of TGase on growth curve and surface of *C. albicans* yeast cells.** *A* and *B*, yeast cells were grown at different concentrations of cystamine (0, 50, 100, and 200 mm) for up to 24 h in rich medium. Additional amounts of cystamine similar to concentrations used at the beginning of the experiment were added to each culture at 6 h (*B*). *Arrows*, addition of fresh cystamine. *C,* in the presence of cystamine, some cells displayed visible fibrillar structures on the surface as visualized by SEM. Moreover, an alteration in division patterns was observed with low frequency (near 2.0% at 50 and 100 mm cystamine; 8.4% at 200 mm cystamine). Remarkably, most cells grown at 200 mm cystamine showed variable numbers of protuberances of different sizes, ranging on average 100 nm in diameter. *Arrows,* alterations in division patterns. *Arrowheads*, protuberances. Statistical analysis, unpaired *t* test: *, *p* < 0.05; **, *p* < 0.01; ***, *p* < 0.001; ****, *p* < 0.0001. *Bars* are shown with standard error of mean.

Growth curves of *C. albicans* cells grown at different cystamine concentrations showed an inhibition of growth cultures as a function of concentration of cystamine. Maximum inhibition was obtained at 200 mm at 6 h of incubation (Fig. 2*A*, *inset*). However, the optical density of cultures was almost the same as the control at 24 h, meaning that the cystamine effect reverted. To test this hypothesis, a second dose of cystamine was added to cultures at 6 h (Fig. 2*B*). A clear effect of cystamine on growth rate was observed and persisted until 24 h of incubation. At this time, the optical density of the culture incubated with 200 mm cystamine was only half the value of the control culture. This means that cystamine transiently inhibits growth rate. Moreover, to determine whether this effect is due to a reversible effect or instability of cystamine, two experiments were carried out previously.^4^ When culture media from yeast cell cultures grown in cystamine with little or no growth were removed and used with fresh cells, cystamine worked properly. However, to analyze a possible instability of the product, fresh media with the different concentrations of cystamine were previously incubated under the same conditions of the experiment for 3 days, and cells were then inoculated, obtaining similar results with those described in this paper.

Cells obtained from the growth curve at 6 h of incubation with different concentrations of cystamine were analyzed by SEM to evaluate possible morphological changes (Fig. 2*A*, *inset*). A population corresponding to 4.3, 3.2, and 4.3% for 50, 100, and 200 mm cystamine, respectively, showed defects in cell separation. Moreover, fibrillar material on the cell surface of cystamine-treated cells appeared on the biggest cells only. They corresponded to 1–2% of cells treated with either 50 or 100 mm cystamine, whereas the affected cells were 8.4% in 200 mm cystamine (Fig. 2*C*). Remarkably, protrusions of ∼100 nm in diameter on the cell surface were visualized in cells treated only with 200 mm cystamine (Fig. 2*C*), which can be extracellular vesicles, cell debris, or small cell wall expansions due to the osmotic shock.

**Figure 3.**
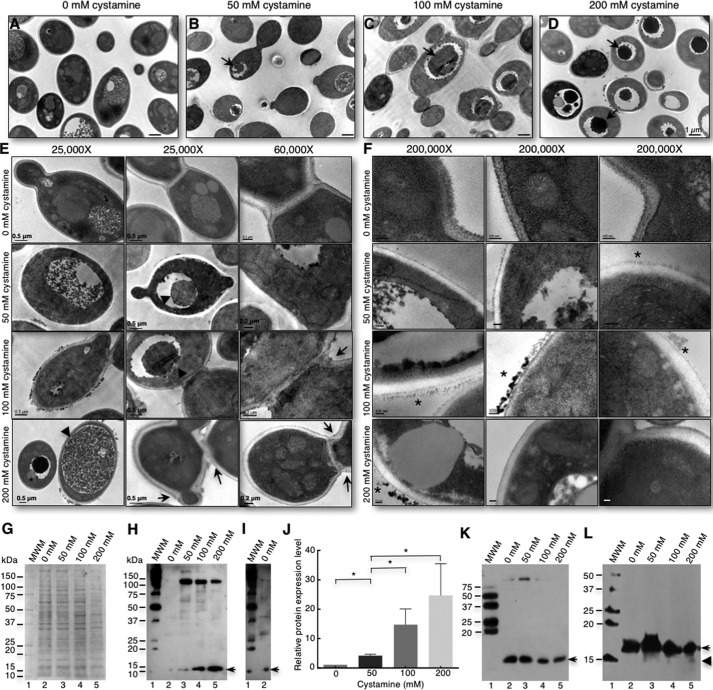
**Inhibition of TGase activity by cystamine induces an increase in autophagy response in *C. albicans* yeast cells.** *A–D*, yeast cells cultured for 6 h in 0, 50, 100, and 200 mm cystamine were harvested and analyzed by TEM. An increase in electron-dense material in vacuoles (*D*, *arrows*) was observed and directly correlated with an increase in cystamine concentration. Furthermore, some cells displayed electron-dense material (*stars*) on the surface of cell walls only when grown in cystamine (*A–F*). *E*, alteration to cell division (*arrows*) was clearly seen in some cells. *F*, magnification of cell wall sections showed loss of density to electrons, and electron-dense material accumulation on the surface. *Scale bar,* 100 nm. *G*, SDS-PAGE of whole-cell extracts obtained from cells grown at 6 h in 0, 50, 100, and 200 mm cystamine. *H*, immunoblot of proteins shown in *G* using anti-LC3A/B antibodies. *Arrow,* 14-kDa band; *MWM*, protein molecular weight markers. *J*, overexposure to chemiluminescence film of *lanes 1* and *2* from Western blot shown in *I. Arrow,* 14-kDa band. *I*, overexposure of *lanes 1* and *2* (control without cystamine) from the Western blot shown in *H. J*, *graph* of relative expression levels of PE-Atg8 protein showing augmented protein in cells treated with increasing concentrations of cystamine. *K*, Western blot of samples electrophoresed through a 12% SDS-polyacrylamide gel containing 6 m urea and revealed with anti-LC3A/B antibodies. *L*, Western blot of samples electrophoresed through a SDS-polyacrylamide gradient gel (4–20%; 15 × 17-cm gel) containing 6 m urea and revealed with anti-LC3A/B antibodies. *, *p* < 0.05 (unpaired *t* test). *Bars* are shown with standard error of mean.

Next, the changes in the ultrastructure of cells were investigated using cells grown for 6 h with 0, 50, 100, and 200 mm cystamine. An accumulation of electron-dense material in vacuoles was observed, which was augmented with increasing amounts of cystamine (Fig. 3, *A–D*, *arrows*). Some vacuoles contained lipid droplets (Fig. 3, *D* and *E*, *stars*), and others occupied most of the cytoplasm (Fig. 3*E*, *200 mm cystamine*). These data suggest an increase in the degradation rate of intracellular material, characteristic of the autophagy process in yeast cells (25). TEM of thin section of cells grown with no TGase inhibitor show the characteristic morphology of yeast cells. When cells were cultured in cystamine-containing medium, an alteration of the division pattern was observed; some cells have two budding sites on opposite poles (Fig. 3*E*, *50 mm cystamine*); other cells budded, but the separation of the mother-daughter cell was not completed. Moreover, some cells began the budding process at other sites (Fig. 3*E*, *100* and *200 mm cystamine*). An analysis at ×200,000 revealed loss of electron-dense material in inner walls when cells were treated with cystamine (Fig. 3*F*, *50, 100,* and *200 mm cystamine*), with accumulation of electron-dense material in the outer wall (Fig. 3*F*, *stars*) compared with control cells. Furthermore, an increase in the number of small vesicles near the plasma membrane was seen. These data suggest that TGase activity inhibition affects the organization of cell wall components and induces increased autophagy in response to the stress caused by cystamine.

To demonstrate the increase in autophagy, we evaluated the level of the LC3/Atg8 autophagy protein marker (25). We performed an alignment of human LC3 and *S. cerevisiae* and *C. albicans* Atg8 homologous proteins to identify the sequence around lysine 40 recognized by the anti-human LC3/Atg8 polyclonal antibody (Fig. S4). This domain in *Candida* Atg8 showed 36.4% identity and 59% similarity to human LC3. Western blot analysis of whole-protein extracts from cells grown in 0, 50, 100, and 200 mm cystamine for 6 h revealed a significant increase in Atg8/LC3 proteins bound to autophagosome membranes (LC3-II, lipid-bound form of LC3 protein with a molecular mass of 14 kDa) with increasing amounts of cystamine (Fig. 3, *G–J*). However, the unmodified Atg8/LC3 16-kDa band was not observed. Interestingly, we also observed a decrease in a 125-kDa band with increasing concentrations of cystamine (Fig. 3*H*). Cells grown without cystamine also showed bands of 14 and 125 kDa when the nitrocellulose membrane was overexposed to chemiluminescence film (Fig. 3*I*). To improve the resolution of the 14- and 16-kDa bands, we performed a Western blot (WB) analysis of samples in a 12% SDS-polyacrylamide gel containing 6 m urea (Fig. 3*K*). We observed the nearly complete disappearance of the 125-kDa band, as well as the presence of a band between 14 and 16 kDa. As we did not observe a good resolution between the 14- and 16-kDa bands in this analysis, we performed a WB assay of electrophoresed samples in an SDS-polyacrylamide gradient gel (4–20%; 15 × 17-cm gel) containing 6 m urea. In this case, all the proteins identified by anti-LC3A/B antibodies were in the 14–16-kDa range. Interestingly, we detected a small amount of the band corresponding to the processed Atg8 polypeptide as a smear, which can indicate that the 16-kDa band can also contain the processed protein. Another important observation is that this smear was not observed in the sample obtained from cells grown without cystamine, indicating no autophagy or very low autophagy. This was confirmed by immunofluorescence with the anti-LC3A/B antibody that showed no autophagy or a very low level of autophagy in normal cells (Fig. S5). Staining was only observed in cystamine-treated cells and was similar when rapamycin was used as an inducer of autophagy. These results confirm the increase in autophagy during TGase activity inhibition by cystamine in *C. albicans* cells.

### Inhibition of TGase activity with cystamine does not affect the amount of chitin, glucan, and mannoproteins in C. albicans yeast cell walls

**Figure 4.**
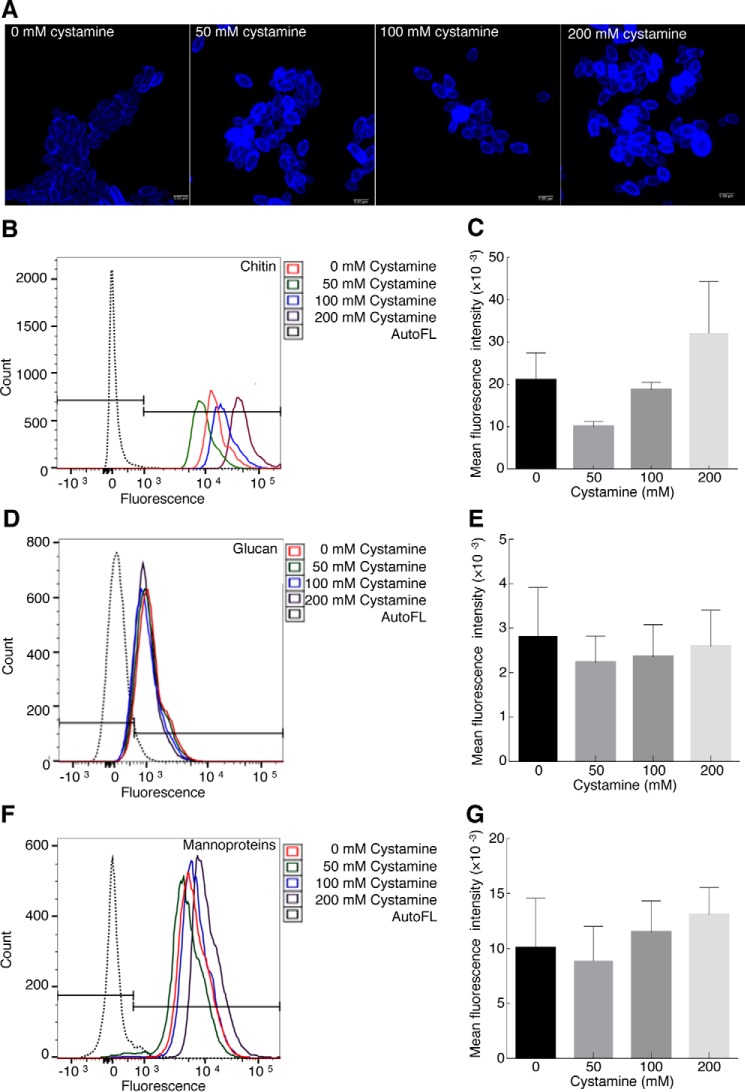
**Inhibition of TGase activity by cystamine does not affect level of cell wall components.** Yeast cells were grown with different concentrations of cystamine (0, 50, 100, and 200 mm) for 6 h at 28 °C, and amounts of mannoproteins, glucan, and chitin were determined by flow cytometry. Chitin staining was first evaluated by fluorescence confocal microscopy using calcofluor (*A*). Chitin (*B* and *C*), glucan (*D* and *E*), and mannoproteins (*F* and *G*) were stained with CFW, methyl blue, and FITC-ConA, respectively. Mean fluorescence intensity was calculated for each experiment and graphed. *Histograms* did not show statistically significant variation in the amount of these components in cells grown at different cystamine concentrations. *AutoFL*, autofluorescence.

We have previously shown changes in yeast cell walls by TEM, as well as the major accessibility of the β-1,3-exoglucanase present in zymolyase for its target when cells were preincubated with different concentrations of cystamine. These results suggest variation in the amounts of different cell wall components. To test this hypothesis, the amounts of chitin, glucan, and mannoproteins were evaluated in yeast cells grown with different concentrations of cystamine for 1–6 h by flow cytometry. Cells were stained more with calcofluor at 200 mm cystamine at 6 h under confocal microscopy (Fig. 4*A*). However, no significant changes were obtained at different culture time intervals. For example, we show the quantitative results for chitin (Fig. 4, *B* and *C*), glucan (Fig. 4, *D* and *E*), and mannoproteins (Fig. 4, *F* and *G*) obtained at 6 h, which are contrary to results obtained by treating *Alternaria infectoria* cells with other antifungal drugs, where changes in the amounts of chitin or glucan were observed using echinocandins and nikkomycin Z, respectively (26).

### TGase activity is essential for the yeast-to-mycelium transition in C. albicans

**Figure 5.**
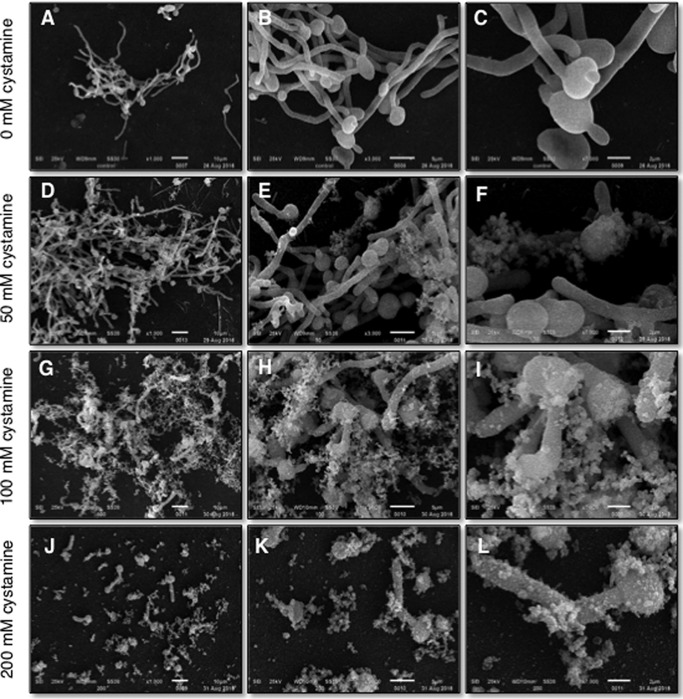
**Inhibition of TGase activity by cystamine severely affects hypha formation.** Yeast cells were grown with (*D–L*) or without (*A–C*) cystamine in LEE medium at 37 °C for 4 h and observed by SEM. Images in *D–L* revealed a decrease in hypha formation and the presence of huge amounts of flocculent extracellular material, which increased at higher concentrations of cystamine.

The conversion from yeast to hyphae has been shown to be essential for the virulence of *C. albicans* (27). It was previously reported that inhibition of TGase activity during myceliation causes the formation of pseudomycelia, short mycelia, and the presence of budding yeasts when 50 mm cystamine was used (23). To characterize in more detail the effects caused by cystamine during the yeast-to-mycelium transition, cells incubated without cystamine showed normal structures during transition (Fig. 5, *A–C*). With increasing concentrations of cystamine, the length of mycelia dramatically diminished in the *C. albicans* CAI4 strain. Notably, the amount of extracellular flocculent material also increased as a function of cystamine concentration (Fig. 5, *D–L*). This effect is similar to that found in response to treatment of *Candida tropicalis* yeast cells with only baicalein or in combination with fluconazole (28).

### Identification of a protein with TGase activity in the cell walls of C. albicans

**Figure 6.**
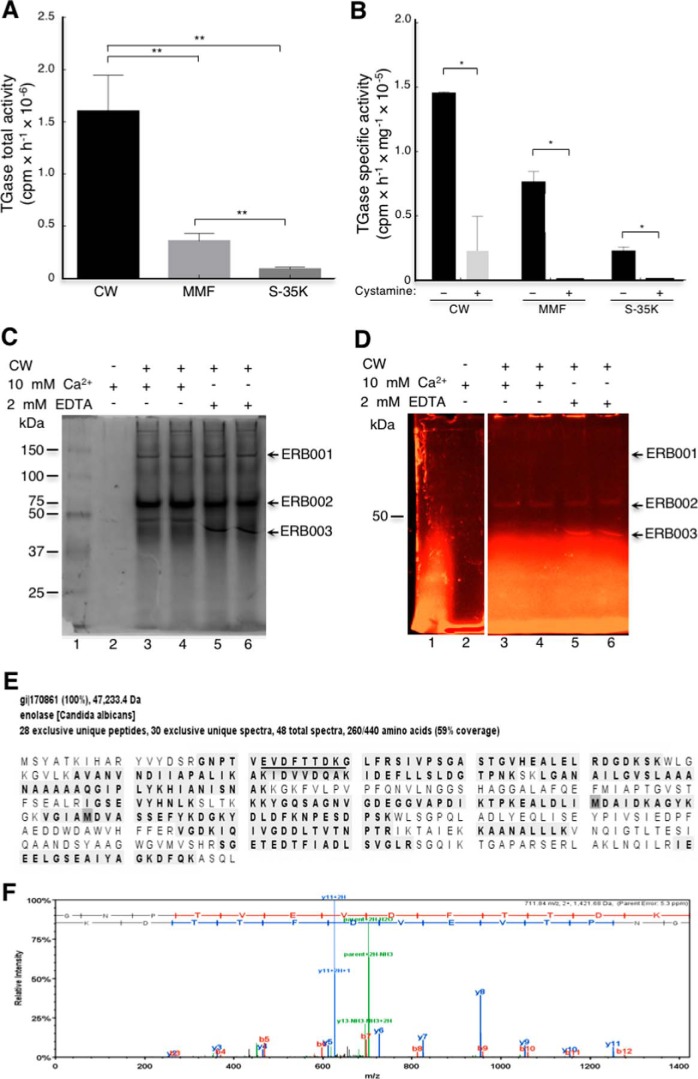
**Determination of TGase activity in cell fractions obtained from *C. albicans* yeast cells and identification of protein with TGase activity.** *A*, total TGase activity was determined by incorporation of [^14^C]lysine in CW, MMF, and cytosolic soluble fraction (S-35K). *B*, specific TGase activity of different cellular fractions and inhibition by cystamine. *C* and *D,* to identify the protein with TGase activity, MDC-specific TGase inhibitor was used to localize the TGase protein by fluorescence in native 7.5% polyacrylamide gels using the cell wall fraction as an enzymatic source. *C*, Gel stained with Coomassie Brilliant Blue. *D,* fluorescent image of gel shown in *C* before staining with Coomassie Blue. A negative control without cell wall was also analyzed in this experiment but is not shown in figure. *Arrows* indicate fluorescent bands isolated for mass spectrometry analysis. *E*, peptides identified by mass spectrometry (28 unique peptides) obtained from ERB003 band in *D* are shown *shaded. F*, tandem mass spectrum of EVDFTTDKG peptide (*underlined* in *E*). Statistical unpaired *t* test. *, *p* < 0.05; **, *p* < 0.01. *Bars* are shown with standard error of mean.

TGase activity has been demonstrated in multiple cellular fractions in *S. cerevisiae* and *C. albicans*, but the identity of the protein responsible for TGase activity has remained elusive (23, 24). To identify this protein, we determined the TGase activity mainly associated with *C. albicans* cell walls (CW, 72%), followed by the mixed membrane fraction (MMF, 19%) and cytosol (S-35K, 9%) (Fig. 6*A*). Activity was reduced 88% in CW fraction, although it was completely inhibited in MMF and S-35K by 50 mm cystamine (Fig. 6*B*). Therefore, we used the CW fraction as the enzymatic source for purification studies. Several approaches were used to solubilize the functional enzyme, including digestions with zymolyase, chitinase, and extractions with 8 m urea, with no results; after centrifugation at 12,000 × *g*, enzymatic activity was always located in the pellet (Fig. S6). Thus, we decided to use MDC inhibitor as a fluorescent probe in a TGase activity assay to identify the protein (Fig. 6, *C* and *D*). The assay was performed with and without Ca^2+^ and with EDTA. This approach allowed us to observe three fluorescent protein bands (Fig. 6*D*) named ERB001, ERB002, and ERB003. The presence of calcium shifted the mobility of the ERB003 band to a higher molecular mass (Fig. 6, *C* and *D*), with no apparent changes for the other two bands. The ERB001 band was tiny and lost its fluorescence very quickly. Identification of proteins in the three fluorescent bands was performed by tandem mass spectrometry (Tables S5 and S6). Interestingly, we identified enolase as a putative candidate TGase enzyme because the most peptides were identified in the ERB002 and ERB003 bands. As an example, we show the peptides identified for enolase corresponding to 59% coverage of the enolase sequence (Fig. 6*E*). These findings corroborate previous results locating enolase in yeast cell walls (29).

**Figure 7.**
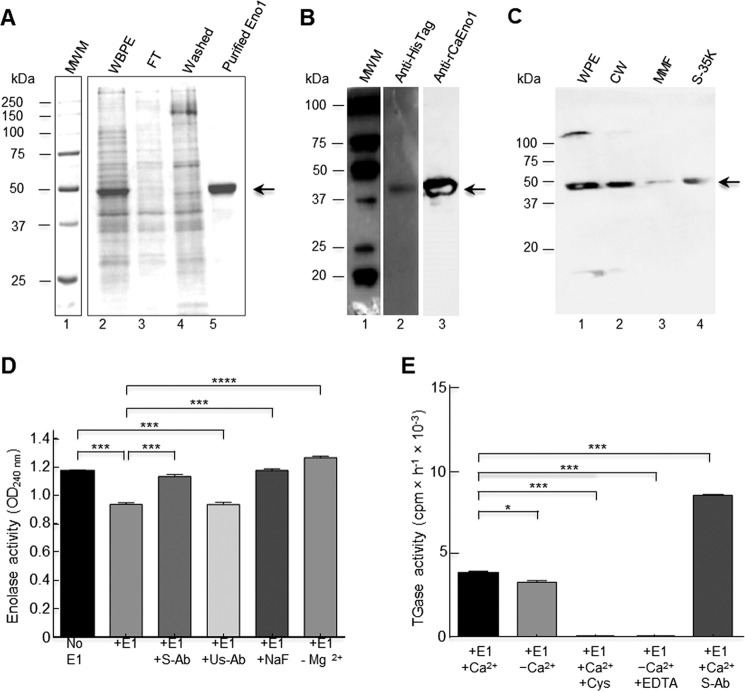
**Recombinant enolase1 from *C. albicans* has TGase activity.** The *C. albicans ENO1* gene was cloned in the pCold II plasmid and transformed into *E. coli* BL21 (DE3) pLysS-competent cells; protein production was induced at 23 °C for 24 h. *A*, Eno1 protein was purified by IMAC with a Ni^2+^-NTA–agarose column in native conditions as described, and elution fractions were evaluated by 12% SDS-PAGE; *MWM*, protein molecular weight markers. Empty vector was also transformed in bacteria and passed through the same IMAC column, and the fractions obtained were also analyzed as a negative control (data not shown). *B*, Western blot of purified recombinant protein using anti-His–tag polyclonal antibodies (*lane 2*) and rabbit anti-rCaEno1 protein (*lane 3*). *C*, Western blot of *C. albicans* cell fractions using anti-rCaEno1 polyclonal antibodies. *WPE*, whole-protein extracts; *CW*, cell wall fraction; *MMF*, mixed membrane fraction; *S-35K*, soluble cytosolic fraction. *Arrows* indicate Eno1 protein. *D*, enolase activity was determined with purified rCaEno1 protein. *E*, TGase activity determined with purified rCaEno1 protein. These results allowed us to conclude that rCaEno1 protein has both enolase and transglutaminase activities. Statistical unpaired *t* test. *, *p* < 0.05; ***, *p* < 0.001; ****, *p* < 0.0001. *Bars* are shown with standard error of mean. *E1,* enolase 1; *S-Ab,* specific antibodies (anti-rCaEno1); *Us-Ab,* unspecific antibodies (anti-rEhPCNA); *Cys,* cystamine.

**Figure 8.**
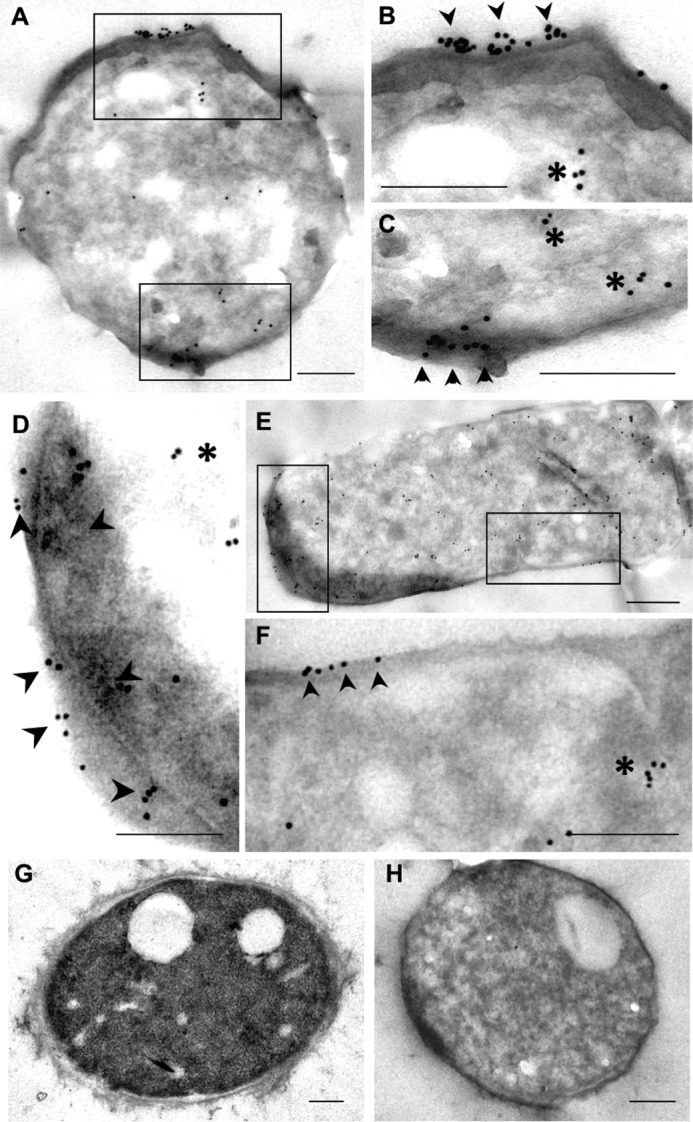
**IEM localization of ENO1 in *C. albicans*.** Micrographs *A–C* and *D–F* correspond to low and high magnifications of two sets of yeasts processed for immuno-gold labeling for detection of ENO1. *G* corresponds to a yeast processed to preserve general ultrastructure. *H* corresponds to the negative control incubated with normal rabbit serum. *Scale bar,* 0.2 μm.

To demonstrate the putative TGase activity of enolase, the gene encoding this protein was cloned into pCold II, and the native recombinant CaEno1 (rCaEno1) produced in *Escherichia coli* BL21(DE3)pLysS was purified by immobilized metal affinity chromatography (IMAC) through a Ni^2+^-NTA–agarose column using 250 mm imidazole (Fig. 7*A*). The identity of the rCaEno1 protein was verified by Western blotting with anti-His–tag antibodies (Fig. 7*B*, *lane 2*) and tandem mass spectrometry (Fig. S7). Purified protein was also used to produce rabbit polyclonal anti-rCaEno1 antibodies, which recognized the purified protein (Fig. 7*B*, *lane 3*), as well as a polypeptide of 47 kDa in whole-cell extracts, purified cell walls, and a mixture of the membrane fraction and soluble fraction of *C. albicans* yeast cells (Fig. 7*C*). Moreover, a 120-kDa band was detected in whole-cell extracts and the cell wall fraction (barely seen). To confirm these results, the distribution of Eno1 in *Candida* yeasts was determined by the IEM technique using the rabbit anti-rCaEno1 polyclonal antibody we generated. Most of the gold labeling was distributed in isolated patches over the surface of yeast (Fig. 8, *A–F, arrowheads*). Gold nanoparticles were also found in the cytoplasm of yeasts (Fig. 8, *A–F, asterisks*). Negative controls with preimmune serum did not show labeling in cells (Fig. 8*H*).

### C. albicans enolase has TGase activity

To demonstrate the TGase activity of enolase, we first demonstrated the enolase activity of rCaEno1. Enolase activity was spectrophotometrically determined by measuring the absorption of phosphoenolpyruvate. When this molecule was transformed in 2-phosphoglycerate, a reduction of 0.2 in the OD_240 nm_ was observed (Fig. 7*D*) demonstrating enolase activity. This was inhibited by the anti-rCaEno1 antibody as well as fluoride ion, a specific inhibitor of enolase (30). As a negative control, we used anti-recombinant *Entamoeba histolytica* proliferating cell nuclear antigen (anti-rEhPCNA) antibodies with no inhibition of enolase activity. This activity was fully dependent on magnesium ions (Fig. 7*D*), as has been reported (31).

TGase activity was determined using the rCaEno1 protein as an enzyme source. Notably, rCaEno1 showed TGase activity in the presence and absence of Ca^2+^ (Fig. 7*E*). It is well-known that TGase activity in *C. albicans* and *S. cerevisiae* is dependent on calcium ions (23, 24). The activity of TGase in the absence of exogenous calcium could be due to the incorporation of this cation in enzymes during expression in bacteria and was conserved through the purification process. To test this hypothesis, we use EDTA to chelate endogenous calcium; consequently, the enzyme showed no activity (Fig. 7*E*). This behavior was similar when TGase activity was tested in 50 mm cystamine. To assess whether the anti-rCaEno1 antibody was also able to inhibit this enzymatic activity, we performed a TGase activity reaction with the protein previously incubated with the anti-rCaEno1 antibody. Surprisingly, this antibody did not inhibit TGase activity but increased it 100% (Fig. 7*E*). These results suggest that there are two different active sites in the enolase molecule.

### TGase activity is essential for growth in several strains of C. albicans

To determine whether TGase activity was necessary for growth in other *C. albicans* strains, we cultured eight strains in solid media containing 0, 25, 50, and 100 mm cystamine (Fig. S8*A*). There were differences in sensitivity to cystamine concentration between yeast strains, with almost complete inhibition for 100 mm cystamine. When 0–3 mm MDC were tested, differences in susceptibility to inhibitor among strains were seen; 3 mm MDC completely inhibited growth in all strains (Fig. S8*B*).

### Cystamine and MDC TGase inhibitors affect the myceliation of C. albicans 26555 strain

**Figure 9.**
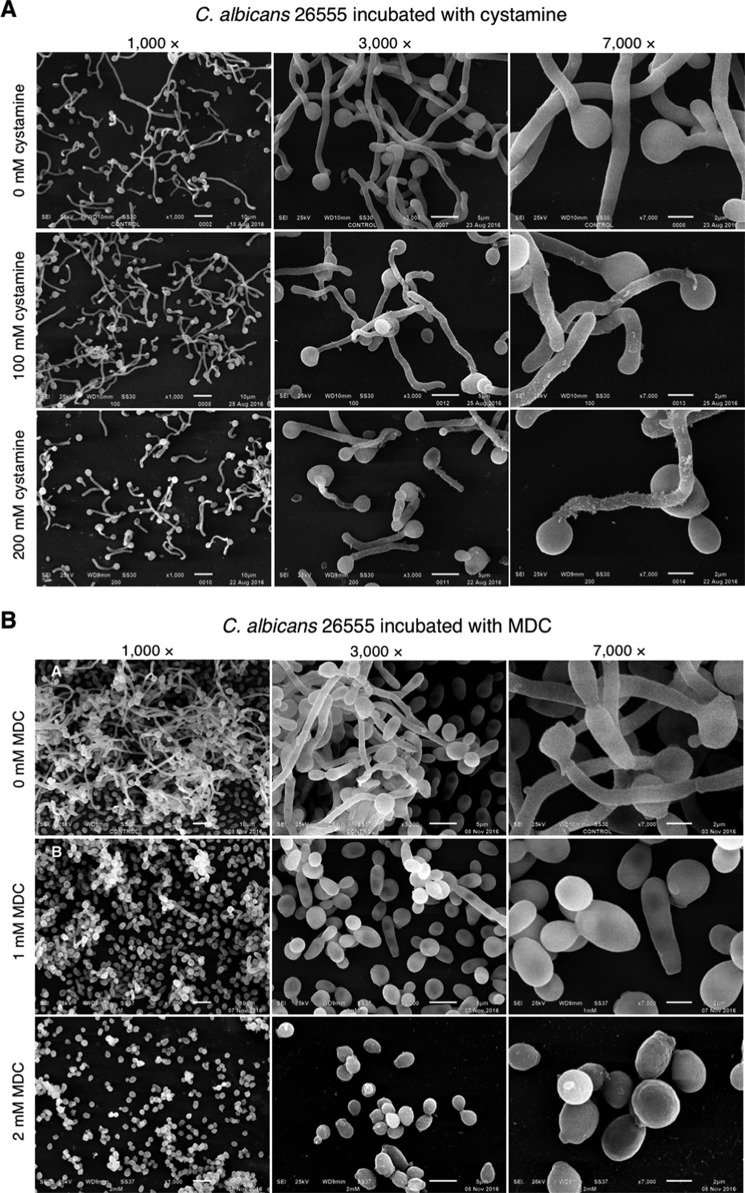
**Effect of cystamine and MDC on yeast-to-mycelium transition of *C. albicans* ATCC 26555 strain.** Different strains of *C. albicans* were grown in YNB medium overnight, and serial 10^−1^ dilutions were used to inoculate YPD solid media containing 0, 25, 50, and 100 mm cystamine (*A*) or 0–3 mm MCD (*B*). Starting concentration of cells used was 3 × 10^6^ cells/ml.

We tested the inhibition of myceliation by both cystamine and MDC TGase inhibitors using the *C. albicans* 26555 strain as an example (Fig. 9). Cystamine inhibited the yeast-to-mycelium transition (Fig. 9*A*), as occurs with the *C. albicans* CAI4 strain. However, the mycelium was longer, and the amount of extracellular flocculent material was lower in *C. albicans* 26555 than *C. albicans* CAI4 (Fig. 5). In addition, MDC completely inhibited the yeast-to-mycelium transition in 2 mm MDC, with no visible damage to its cell surface (Fig. 9*B*).

## Discussion

*C. albicans* is the main opportunistic pathogenic fungus in immunocompromised patients worldwide (32). The principal structure of *C. albicans* in contact with host cells is the cell wall, which mainly confers osmotic protection, cellular shape, and defense against host immune response. Cell wall components are cross-linked and provide stability to this structure through different types of chemical bridges. TGase is involved in enzymatic activities in cell walls and the formation of covalent cross-links previously demonstrated in *S. cerevisiae* and *C. albicans* (23, 24). Inhibition of TGase by cystamine and MDC, which are irreversible and reversible inhibitors (33–35), respectively, increased the osmosensitivity of cells after partial digestion of glucan with zymolyase (Fig. 1, *D* and *E*). A decrease in the optical density at 600 nm of cultures with inhibited TGase indicated that the protective role of the cell wall against osmotic shock was diminished as a result of the easier access of glucanase from zymolyase to glucan. It is well-known that the hydrolysis of glucan by lyticase or zymolyase (two commercial preparations of glucanases) causes the formation of protoplasts or spheroplast, which are highly sensitive to osmotic shock. Thus, TGase catalyzed the formation of isopeptide chemical bonds between the [^14^C]lysine and proteins of both low- and high-molecular masses (Fig. 1*C*) to bring osmotic protection to cells.

The mass spectrometry analysis of the labeled proteins released with SDS revealed a great diversity of proteins (1046) with different functions in the cell, including glycolysis, mitochondrial functions, translation, heat shock stress, membrane proteins, structural proteins, nucleic acid-binding proteins, among many others. The average molecular masses of these proteins were below 50 kDa; however, there were many proteins with high-molecular masses (between 50 and 220 kDa). Because the range of molecular masses of proteins in the gel that we cut for mass spectrometry analysis was lower than 50 kDa, these proteins then might not have their full length in the cell wall, and their fragments could have been cross-linked to other components of the cell wall by the transglutaminase. The number of proteins released with zymolyase and chitinase was lower (37 and 41, respectively). The proteins released by zymolyase were mainly ribosomal subunits, glycolytic proteins, heat-shock proteins, and six cell wall proteins, which correspond to agglutinin-like protein 1 (Q5A8T4), agglutinin-like protein 4 (A0A1D8PQB9), chitinase 2 (P40953), the covalently-linked cell wall protein 14 (Q5AFN8), the cell-surface Cu-only superoxide dismutase (Q5AD07), and the pH-responsive protein 2 (O13318), which has 1,3-β-glucanosyltransferase activity. Interestingly, their molecular average masses were between 16 and 268 kDa, indicating that they are cross-linked to glucan or other cell wall components to ensemble large complexes. The proteins released with chitinase were similar to those released with zymolyase and might be linked to chitin or other cell wall components. More studies are needed to determine the role of these proteins identified in the cell wall. Recently, the molecular composition of extracellular vesicles (EVs) isolated from *C. albicans* was published (36), finding some similar proteins as those described in our work. Some of them included proteins involved in oxidation/reduction, metabolism, translation, stress, transporter, signaling pathways, and mitochondrial function. However, their function in EVs remains unknown as well.

The inhibition of TGase by cystamine affected the growth of yeast cells as a function of inhibitor concentration (Fig. 2). Damage caused by this inhibition included changes to the cell division pattern (Figs. 2*C* and 3*E*) and cell wall ultrastructure because of the changes observed by SEM (Fig. 2*C*) and TEM (Fig. 3, *E* and *F*). The presence of fibrillar material apparently linked to the cell wall was seen on the cell surface of the biggest cells only (Fig. 2*C*). In addition, the external layer of the cell wall seems to be absent in these cells and can correspond to mannoproteins, as these molecules are located at the outermost layer of the cell wall (9, 10, 12). We also observed the accumulation of electron-dense compounds on the surface of cell walls (Fig. 3, *E* and *F*), which seems to be different to the fibrillar material observed by SEM. However, these differences could be due to the different techniques used.

The increase in electron-dense material in vacuoles (Fig. 3, *A–D*) could be suggestive of autophagy, as confirmed by an increase in the Atg8 autophagy marker with increasing concentrations of cystamine (Fig. 3, *G* and *J*) and by the presence of the modified form of Atg8 seen as a smear ranging between 14 and 16 kDa (Fig. 3*L*). The processed form of Atg8 was not observed in control cells even though we used SDS-PAGE gradient gels with 6 m urea. The 125-kDa band detected in Western blottings that decreased with increasing amounts of cystamine (Fig. 3*H*) could correspond to the Atg8 protein associated with other proteins into a complex that was not disrupted with Laemmli buffer (Fig. 3, *G–J*). However, this complex was almost completely disrupted when the SDS-polyacrylamide gel contained 6 m urea (Fig. 3, *K* and *L*). This electrophoretic analysis also allowed the observation of the 16-kDa band in the control sample, which was not previously observed, suggesting that the epitope that is recognized by the antibody was not exposed even though we used Laemmli buffer. This observation was also confirmed when we performed the detection of the Atg8 protein by immunofluorescence analysis (Fig. S5).

Moreover, the noteworthy inhibition of TGase did not affect the amounts of cell wall components, *i.e.* chitin, glucan, and mannoproteins at 6 h of incubation with cystamine (Fig. 4), which also remained unchanged at the previous time points of incubation with cystamine, but their organization might be altered, as observed by TEM (Fig. 3*F*). Notably, the growth inhibition caused by cystamine and MDC was observed in different *C. albicans* strains that displayed variation in sensitivity to inhibitors (Fig. S8), thus confirming the importance of TGase in a number of *C. albicans* strains.

Another process that is considered essential for *C. albicans* pathogenicity is the yeast-to-mycelium transition (37). Our results showed that TGase activity is required for this transition. Notably, the inhibition of TGase activity in the *C. albicans* CAI4 strain was also observed for *C. albicans* ATCC 26555 (Figs. 5 and 8). However, there were differences in the response of cells to inhibition. The CAI4 strain showed more secreted flocculent material than the ATCC 26555 strain, as well as more and longer mycelia. These differences might be attributed to the deletion of the *Ura3* gene in the CAI4 strain, which is an essential gene in myceliation (38). This mutation also affected the cytosol expression levels of Ura3p, Hpt1p, Ald5p, Sgt2p, Pmm1p, IPF6037, Aro8p, Ade2p, Ura5p, Eft2p, Aro10p, Hem13p, Rps12p, Toa2p, and IPF4328 (39). These proteins have different roles in metabolism. Hpt1p, Ade2p, and Ura5p are involved in purine and pyrimidine metabolism. Hem13p and Ald5p are involved in heme biosynthesis, whereas Aro8p and Aro10p play a role in the turnover of aromatic amino acids. Finally, Rps12p, Eft2p, and IPF6037p participate in transcription or translation (39). Although the virulence of cystamine-treated cells has not been examined, it is expected that virulence will be affected because *C. albicans* that are unable to form filaments are not virulent (37).

The identification of CaEno1 as a protein with two functions is not unique. Enolase has been described as a multifunctional protein that also exhibits non-glycolytic functions in several species. The human α-enolase is considered a moonlighting protein because it has many functions, including a role as a neurotrophic factor, as a stress protein involved in hypoxia, heat-shock proteins, or as part of centrosomes (40). In addition, it has binding capacities for cytoskeleton proteins such as F-actin and tubulin (41). Moreover, it interacts with APIP (APAF1 interacting protein), MRI1 (methylthioribose-1-phosphate isomerase yeast homologue), ADI1 (acireductone dioxygenase 1), ADD2 (adducin 2β), ADD1 (adducin 1α), ADD3 (adducin 3γ), SMS (spermine synthase), and UBA6 (ubiquitin-like modifier-activating enzyme 6) (String database). CaEno1 interacts with several proteins of the glycolytic pathway in *S. cerevisiae*, including PGI1 (phosphoglucose isomerase), TPI1 (triose-phosphate isomerase), CDC19 (pyruvate kinase), TDK1 (glyceraldehyde-3-phosphate dehydrogenase 1), TDK2 (glyceraldehyde-3-phosphate dehydrogenase 2), TDH3 (glyceraldehyde-3-phosphate dehydrogenase isozyme 3), PYK2 (pyruvate kinase), PGK1 (3-phosphoglycerate kinase), GPM1 (tetrameric phosphoglycerate mutase), and ENO2 (enolase II) (String database). The *S. cerevisiae* enolase has an additional function as a chaperone bound to preMSK1p for importing tRNA to mitochondria (42) The *C. albicans* enolase 1 interacts with Cbk1 (Cell wall Biosynthesis Kinase 1) (BioGRID database) (41), a Ser/Thr kinase that belongs to the RAM (Regulation of Ace2p transcription factor and polarized Morphogenesis) signaling network involved in polarized growth, cell separation morphogenesis, and biofilm formation (43–47).

Furthermore, enolase is considered the major antigen in patients with candidiasis and confers an advantage for invasion when bound by plasmin to induce fibrinolysis (48, 49). The null mutant was more susceptible to several drugs, did not show hyphal growth, and had reduced virulence in mice (50). In *S. cerevisiae*, the recombinant enolase causes vacuole fusion *in vitro*, and the null mutation of this gene generates vacuole fragmentation as a result of reduced vacuole fusion (51).

The CaEno1 and ScEno1 polypeptides have 78% identity and 88% similarity in the 436 compared amino acids (Fig. S9). The CaEno1 structure was obtained by homology modeling with the Modeler program, using the structure of ScEno1 (PDB code 2al1) as a template (Fig. S10), and it had good quality as shown by Verify3D (98.40% of residues had an averaged 3D–1D score of ≥0.2), Errat (overall protein quality factor 89.744), and Ramachandran plot (Fig. S11). CaEno1 is classified by the CATH database as a member of the enolase superfamily with the code 3.20.20.120, which indicates that CaEno1 is a protein mainly containing α-helices and β-strands and has a TIM-type α–β-barrel. The similarity between the two proteins was evidenced by superimposing both structures (Fig. S10*C*). In addition, they have identical catalytic residues, as well as residues involved in binding the Mg^2+^ ions and substrate (Fig. S12). However, the electrostatic potential of the CaEno1 surface has a negative value, whereas that of ScEno1 has a more positive surface value (Fig. 10, *D* and *E*). This may produce differences in proteins that interact with the two proteins. Because of the similarity of these two proteins, ScEno1 might also have transglutaminase activity, as it localizes in yeast cell walls that also have TGase activity (24).

Our data indicate that enolase does not share the same catalytic site for enolase and TGase enzymatic activities, because anti-rCaEno1 antibodies only inhibited enolase activity. Instead, it favored the transamidation reaction (Fig. 7, *D* and *E*) due to putative conformational changes in the protein, as demonstrated for human TG2. In this case, the conformational changes are involved in controlling enzymatic activity and are important for regulating the enzymatic activity of autoantibodies in celiac disease (52).

An alignment with Clustal Omega program showed that CaEno1 has an identity of 18.72, 15.54, 18.59, 17.36, 16.85, and 20.61% with transglutaminases from *Anopheles gambiae*, *Mus musculus*, *Gallus gallus*, *Apis mellifera*, and *Homo sapiens* (TGase and Factor XII), respectively. However, the similarity increases in some conserved boxes (Fig. S13). Even though the identity is low, we identified a putative TGase catalytic site (AFQEFMIAPTGVSTFSEALR) in CaEno1, which is shown underlined in Fig. S13, and it corresponds to the PROSITE PS00547 identifier with the sequence (GT)Q(CA)WV*X*(SA)(GAS)(IVT)*X*(2)T*X*(LMSC)R(CSAG)(LV)G (http://prosite.expasy.org/PS0054).^5^ This putative TGase catalytic site was localized in the CaEno1 protein structure (Fig. S14). Interestingly, the Glu-170 residue in this sequence is part of the catalytic site of enolase (Fig. S12). Other residues (AFQEFMIA) are located in a β-sheet, which is localized close to the enolase active site. The remaining residues are located in an α-helix. This could suggest that the TGase active site needs a conformational change to be functional, depending on pH and the presence of calcium ions. There have been described two conformations for human TG2, open and closed. The presence of GTP without Ca^2+^ induced the closed conformation of TG2 with no TGase activity; when Ca^2+^ is present only, the TG2 conformation is open and therefore the protein is active (52). However, the answer for CaTGase will be revealed by the X-ray structure determination in the conditions used for the measurement of the activity of enolase and/or transglutaminase.

In conclusion, the aforementioned data spotlight TGase as a key molecule involved in cell wall remodeling during cell division and yeast-to-mycelium transition and as a putative molecular target for designing new drugs to specifically attack this important medical fungus.

## Experimental procedures

### C. albicans strains and culture conditions

We used the following *C. albicans* strains: *C. albicans* ATCC 26555; *C. albicans* CAI4 (ura3/ura3Δ:: imm434) (53); *C. albicans* 1392; *C. albicans* 1394; *C. albicans* 1002; *C. albicans* 1675; *C. albicans* 1676; *C. albicans* 1687; and *C. albicans* 1439. The last seven strains were obtained from the Colección Española de Cultivos Tipo. All yeast strains were grown in liquid or solid YPD (1% yeast extract, 2% Bacto peptone, 2% glucose) or YNB (Gibco) at 28–30 °C. For *C. albicans* CAI4, YPD medium was supplemented with 25 μg/ml uridine.

To study the effects of cystamine on growth, *C. albicans* CAI4 yeast cells were previously grown in YPD medium (1% yeast extract, 2% Bacto peptone, 2% glucose) supplemented with uridine (25 μg/ml) at 30 °C overnight at 200 rpm in a water bath incubator (New Brunswick Scientific). Aliquots were withdrawn and inoculated in each of four Erlenmeyer flasks containing 100 ml of fresh media with different concentrations of cystamine (0, 50, 100, and 200 mm) to an initial optical density of 0.1 at 600 nm and incubated at 30 °C and 200 rpm for 24 h. During incubation, aliquots were withdrawn several times to determine the OD of cultures. For the inhibition experiments of other *C. albicans* strains, the same protocol was used, excluding uridine. Alternatively, MDC was used at different concentrations (0–4 mm).

### Induction of myceliation in C. albicans

Yeast cells were grown in Lee's medium (23, 24) supplemented with 25 μg/ml uridine for the *C. albicans* CAI4 strain, with shaking at 200 rpm and 30 °C for 16 h. The cells were harvested by centrifugation, washed twice with distilled water, resuspended at a concentration of 200 μg/ml (dry weight) in sterile deionized water, and kept at 4 °C for 48 h. Adequate volumes of cells were used to inoculate fresh Lee's medium (OD_600 nm_ = 0.2) and were incubated with different concentrations of cystamine (0, 50, 100, and 200 mm) at 37 °C and 200 rpm for 4 h to inhibit myceliation. In some cases, yeast cells were also incubated in 0, 1, and 2 mm MDC at 37 °C and 200 rpm for 4 h. Finally, cells were analyzed using a scanning electron microscope from the National Laboratory of Experimental Services (LaNSE) at Cinvestav-IPN.

### Fractionation of yeast cells

Cell-free extracts were obtained by mixing cells with 4 g of glass beads (0.5 mm in diameter, BioSpec Products) per g of cell pellet and were broken by vigorous agitation in buffer A (50 mm Tris-HCl, pH 7.4, containing 1 mm phenylmethylsulfonyl fluoride (PMSF) and 1 μg/ml pepstatin A) for periods of 30 s on a vortex mixer and cooling periods of 1 min in ice until all cells were completely disrupted. Extracts were centrifuged at 1200 × *g* at 4 °C in a 5810R Eppendorf centrifuge, and sedimented walls were washed twice in 50 mm phosphate buffer (pH 7.4), twice with 2 m NaCl, and twice with deionized water and then kept on ice. The supernatant was centrifuged at 105,000 × *g* in a 90 Ti rotor in a Beckman Coulter Optima TM L-100 XP Ultracentrifuge for 1 h at 4 °C to obtain the soluble fraction (S-35K) and the mixed membrane fraction (MMF) and processed immediately to determine TGase activity.

### Sequential extraction of cell wall proteins with SDS, zymolyase, and chitinase

Purified cell walls, previously labeled with [^14^C]lysine, were sequentially extracted with SDS, zymolyase (Miles Laboratories), and chitinase (Sigma) as described (24, 54). Briefly, the treatment with 2% SDS was performed in a boiling water bath for 10 min, and solubilized proteins were recovered by centrifugation at 3000 × *g* for 10 min at room temperature. The remaining insoluble material was washed by centrifugation with water, ethanol, and water, and this process was repeated. The material was treated with 1 mg/ml zymolyase 20T containing 1 mm PMSF at 30 °C for 3 h, and solubilized material was separated by centrifugation. Finally, the pellet was treated with 0.5 mg/ml chitinase in 10 mm phosphate buffer, pH 7.0, at 30 °C for 3 h, and solubilized material was collected. Radioactivity in all samples was quantified as above. Samples extracted with SDS were precipitated with 75% ethanol at 4 °C and washed three times with ethanol by centrifugation. Samples containing 10,000 cpm were electrophoresed in 10% SDS-polyacrylamide gels, stained with Coomassie Brilliant Blue, incubated with Amplify solution (GE Healthcare) as recommended, dried, and exposed to Kodak X-Omat S films at −70 °C for 1 month (24).

### Paper chromatography

Purified radioactive cell walls were sequentially solubilized with 2% SDS, 1 mg/ml zymolyase, and 0.5 mg/ml chitinase, as described previously. The released proteins were separated from insoluble residue by centrifugation at 3000 × *g* for 10 min at room temperature, and radioactivity was quantified. Samples containing 10,000 cpm were analyzed by electrophoresis in 10% SDS-polyacrylamide gels and fluorography. Alternatively, samples were analyzed by paper chromatography in saturated phenol with water into an ammoniac atmosphere. To determine the distribution of radioactivity, paper chromatograms were cut into 1-cm strips to determine radioactivity in a liquid scintillation counter (see below).

### Determination of enzymatic activities

For the initial measurement of transglutaminase activity, 2.5 μCi of [^14^C]putrescine (specific activity 108 mCi/mmol) or 2.5 μCi of [^14^C]lysine (specific activity 9.2 mCi/mmol) were used in reaction mixtures containing 1% (w/v) *N,N*′-dimethyl casein, 50 mm Tris-HCl, pH 7.4, and the enzymatic source in a final volume of 1 ml. A commercial TGase from guinea pig liver (Sigma) was used in some assays. Later on, aliquots of different cellular fractions were used to determine the transglutaminase activity in reaction mixtures containing 2 mm CaCl_2_, 0.25 μCi [^14^C]lysine (326.0 mCi/mmol, Matrix Laboratories) in a final volume of 0.25 ml of buffer A. Following 2–6 h of incubation at 30 °C with shaking, reactions were stopped by adding 2 ml of 10% (w/v) TCA, kept on ice for 2 h, filtered through glass fiber filters (2.5 cm in diameter, Whatman) using a vacuum filtration manifold (Millipore), washed twice with 2 ml of cold 5% TCA, twice with 2 ml of cold 70% ethanol, and dried at room temperature. Finally, radioactivity was measured in glass vials containing 5 ml of Ready Protein Liquid Scintillation Mixture (Beckman) and a Beckman LS6000 SC liquid scintillation counter.

Enolase activity was determined spectrophotometrically by measuring the reduction of absorbance at 240 nm of phosphoenolpyruvate (PEP) due to conversion into 2-phosphoglycerate (30). The reaction mixture contained 50 mm imidazole, pH 6.8, 400 mm KCl, 1 mm PEP, and 3 mm MgSO_4_ in a final volume of 200 μl. The activity was evaluated by measuring changes in absorbance at 28 °C for 1 min after the addition of purified recombinant enolase. A decrease in absorbance of 0.2 corresponds to the conversion of 0.226 μmol of substrate (30). For inhibition studies of enolase, we used anti-recombinant *C. albicans* Eno1 (rCaEno1) antibodies and anti-recombinant rEhPCNA antibodies (see below).

### Procedure followed to solubilize TGase activity from cell walls

Cell walls were double-digested with zymolyase and chitinase for 4 h at room temperature, followed by denaturing with 8 m urea and subsequent renaturation by sequential dialysis with 5 and 2 m urea, and 50 mm Tris-HCl, pH 7.4, each 12 h at 4 °C. Sample was then centrifuged at 1200 × *g* for 10 min in an Eppendorf centrifuge at 4 °C; the pellet was discarded, and supernatant (named SN 1200 × *g*) was centrifuged at 12,000 × *g* for 30 min at 4 °C. Pellet and supernatant (SN 12,000 × *g*) were separated and kept on ice. Pellet was resuspended in 50 mm Tris-HCl, pH 7.4 (Pellet 12,000 × *g*) (Fig. S1). Finally, TGase activity was determined in CW, the two SN and final pellet as described.

### SEM

Yeast cells were grown in the absence or presence of different concentrations of cystamine for 6 h as described, harvested by centrifugation, washed three times with 1× PBS, and fixed with 2.5% glutaraldehyde in 1× PBS for 1 h. After fixation, small drops of samples were placed on specimen supports coated with concanavalin A (Sigma) and postfixed with 2% osmium tetroxide in 1× PBS for 1 h. Preparations were gradually dehydrated in different concentrations of ethanol (50, 60, 70, 80, and 90%) for 10 min each, and three times in 100% ethanol for 15 min each. Next, samples were submitted to critical point drying with CO_2_ in a Samdry-780 apparatus (Tousimis Research, Rockville, MD) and metallization in a Denton Vacuum Desk II (Denton Vacuum, Morestown, NJ). Finally, cells were observed through a Jeol JSM-6510LV scanning electron microscope from LaNSE at Cinvestav-IPN.

### TEM

Cells were fixed for at least 1 h in 3% glutaraldehyde in 1× PBS at room temperature. Samples were washed three times with 1× PBS each for 10 min, placed in a 1% osmium tetroxide solution in 1× PBS for 2 h at room temperature, washed thoroughly with water, and stained with 0.1% uranyl acetate overnight at room temperature. Next, samples were dehydrated with different concentrations of ethanol (50, 60, and 70%) for 10 min each, and then with 80, 90, and 100% ethanol for 15 min each. As propylene oxide was used as a transitional solvent, it was changed twice, each time for 10 min. Samples were placed in a 1:1 mixture of DER 736 epoxy resin and absolute ethanol for 3 h at room temperature, and in a 3:1 epoxy resin/ethanol mixture overnight at room temperature. The next day, samples were transferred to a 100% epoxy resin and incubated for 2 h at room temperature, placed in embedding molds, and polymerized for 24 h at 60 °C. Thin sections were cut using a Leica EM UC7 ultramicrotome and a glass knife. Thin sections (60–80 nm) on copper grids were stained with 1% uranyl acetate and Reynold's lead citrate. They were analyzed through a JEOL 1400 transmission electron microscope from LaNSE at Cinvestav-IPN.

### Calcofluor staining of chitin for confocal microscopy

Yeast cells (10 million), obtained from the growth curve at 6 h of incubation as described, were fixed in 70% ethanol for 15 min, washed twice with 1× PBS for 30 s each, incubated with 100 μg/ml CFW (Sigma) for 5 min, washed twice with distilled water, and observed with a DAPI-compatible filter set in a Leica TCS SP8 confocal microscope (Leica Microsystems, Mexico City, Mexico).

### Quantification of chitin, mannoproteins, and glucan by flow cytometry

To measure the amount of chitin (55), 1.5-ml aliquots of cultured cells grown in YPD medium with 0, 50, 100, and 200 mm cystamine at an initial OD_600 nm_ of 0.2 were harvested (1.5 ml) at 1-h intervals up to 6 h by centrifugation at 500 × *g* in an Eppendorf microcentrifuge at 4 °C, washed twice with sterile distilled water, permeabilized with 70% ethanol, washed twice with distilled water, and stained without (to measure autofluorescence) or with 50 μg/ml CFW for 15 min at room temperature. One million cells were washed as before and analyzed through a flow cytometer (BD LSRFortessa from LaNSE at Cinvestav-IPN) to quantify CFW fluorescence (using a DAPI-compatible filter set). In each experiment, fluorescence emitted by 20,000 cells was determined using simple gated forward-scattered light *versus* side-scattered light parameters. The mean intensity of fluorescence emitted for stained (positive population) or no-stained (autofluorescence or negative population) yeast cells was analyzed and processed with FlowJo 10.0.6 software (Tree Star, Inc., Ashland, OR).

For quantification of mannoproteins, staining of cells with fluorescein-labeled concanavalin A (FITC-ConA, Vector Laboratories) was performed at the same time intervals as described previously. Cells were washed twice with 1× PBS, permeabilized with 70% ethanol, incubated with 2 μg/ml FITC-ConA in 1× PBS for 30 min at room temperature, washed twice with 1× PBS, and analyzed in the flow cytometer. The maximum excitation wavelength was 495 nm, and the maximum emission wavelength was 515 nm.

Glucan content was determined as follows: cells were washed twice with 1× PBS, permeabilized with 70% ethanol, washed twice with 1× PBS, and incubated in 0.5 mg/ml methyl blue (Sigma) for 5 min at room temperature. The cells were then analyzed by flow cytometry using an excitation wavelength of 410 nm and emission wavelength at 455 nm.

In each experiment, the staining index (SI) was calculated as shown in Equation 1 (56),

(1) $$\text{SI}=\frac{\left(M_{1}-M_{2}\right)}{2}\;\cdot \;\text{S}$$

where *M*_1_ is the mean fluorescence intensity of the positive population; *M*_2_ is the mean fluorescence intensity of the negative population, and S is the standard deviation of the mean fluorescence intensity of the negative population.

### Western blotting

For *C. albicans* Atg8 immunodetection, we used the LC3A/B rabbit polyclonal antibody (Cell Signaling Technology), which recognizes LC3-I and LC3-II (16 and 14 kDa, respectively). Yeast cells were grown in different concentrations of cystamine for 6 h; yeast cell walls were separated by centrifugation, and remaining supernatants were electrophoresed in 12% SDS-polyacrylamide gels and transferred to nitrocellulose membranes (GE Healthcare, Mexico City, Mexico). Membranes were blocked with 5% skim milk (Difco) in 0.05% Tween 20 in 1× PBS (PBS-T) for 4 h at room temperature and incubated with 1:250 dilutions of LC3A/B primary antibody in 5% skim milk in PBS-T overnight at 4 °C. Next, membranes were washed 10 times with PBS-T for 10 min each at room temperature and incubated with a 1:20,000 dilution of goat anti-rabbit IgG antibody (Zymed Laboratories Inc.) in 5% skim milk in PBS-T for 2 h at room temperature. After washing membranes as described, immunoreactivity was detected by chemiluminescence with a 1:8 dilution of the SuperSignal West Femto maximum sensitivity substrate kit (Pierce, Mexico City, Mexico) using High Performance Chemiluminescence Films (GE Healthcare, Mexico City, Mexico). Digital images of Western blottings were obtained with a Gel Doc EZ Imager system and processed with Image Lab software (both from Bio-Rad). As molecular mass markers, we used Strep-tagged Precision Plus Protein All Blue Standards (Bio-Rad), which were visualized by chemiluminescence with the Precision Protein StrepTactin-HRP conjugate (Bio-Rad). For the identification of rCaEno1 protein, we used either mouse anti-His–tag monoclonal antibodies (1:5,000 dilution; Roche Applied Science) or rabbit anti-rCaEno1 polyclonal antibodies (1:20,000 dilution), following the procedure described above.

### Identification of TGase protein using a fluorescent TGase inhibitor MDC probe

Cell walls from *C. albicans* were simultaneously digested with 60 units of chitinase (Sigma) and 100 units of zymolyase (Zymo Research) overnight at room temperature. This fraction was used as the enzymatic source in the TGase activity assay. Digested cell walls (200 μl) were mixed with 0.2 mm MDC, 1 mm PMSF, 1 μg/ml pepstatin A, with no CaCl_2_ or EDTA, and with 10 mm CaCl_2_ or 2 mm EDTA, and incubated for 1 h at 4 °C. Proteins were separated by non-denaturing 7.5% polyacrylamide gels, and fluorescence was visualized at 350 nm with the Gel Doc EZ Imager system (Bio-Rad). Finally, fluorescent bands were excised and submitted to mass spectrometry (MS/MS) analysis for protein identification to the Proteomics Division at the University of Florida Interdisciplinary Center for Biotechnology Research.

### Expression of recombinant C. albicans Eno1 protein and generation of anti-rCaEno1 polyclonal antibodies

Full-length *CaENO1* gene (1323 bp) flanked by KpnI and BamHI restriction sites was amplified by PCR using genomic DNA using the following primers, CaENO1pColdII_Fw(5′-CGGGGTACCATGTCTTACGCCACTAAAATCCA-3′) and CaENO1pColdII_Rv(5′-CGCGGATCCTTACAATTGAGAAGCCTTTTGGAA-3′), and cloned into the pJET1.2/blunt vector (Thermo Fisher Scientific). It was then subcloned into the pColdII (Takara) plasmid in the mentioned restriction sites to generate the pColdII/CaEno1 plasmid, which was transformed into *E. coli* BL21 (DE3)pLysS (Invitrogen) competent cells. The induction of recombinant CaEno1 protein (rCaEno1) was achieved with 0.5 mm isopropyl β-d-thiogalactopyranoside for 24 h at 23 °C in Luria-Bertani medium at 200 rpm. Cells were harvested by centrifugation at 4000 × *g* for 20 min and resuspended in 12 ml of buffer A (50 mm Tris-HCl, 300 mm NaCl, pH 7.4) containing 1 mm phenylmethylsulfonyl fluoride (PMSF), 1 μg/ml pepstatin A, 10 mm imidazole, and 1 mg/ml lysozyme. Cells were incubated at 4 °C for 1 h and sonicated three times in ice for 10 s at 60% amplitude in a CPX 130PB Ultrasonic Processor (Cole-Parmer, Verno Hills, IL) with cooling for 1 min in ice between each sonication cycle, and the cell-free extract was obtained by centrifugation at 10,000 × *g* for 30 min at 4 °C. Purification of the rCaEno1 protein was performed by immobilized metal affinity chromatography (IMAC) using a Ni^2+^-NTA–agarose column (7.0 × 0.9 cm, 1 ml gel, Sigma). The column was washed with 4 volumes of buffer A, and the rCaEno1 protein bound to the column was eluted with 4 volumes of buffer E (50 mm Tris-HCl, 250 mm imidazole, pH 7.4) at 4 °C, collecting 1-ml fractions.

The identity of the purified rCaEno1 polypeptide was verified by Western blotting and tandem mass spectrometry with a 4800 MALDI-TOF/TOF mass spectrometer (Applied Biosystems) from LaNSE (Cinvestav-IPN). The obtained MS/MS spectra were compared against the *C. albicans* SC5314 (ATCC MYA-2876) database uploaded from UniProt Knowledgebase using the Protein Pilot software version 4.0 (SCIEX, Mexico City, Mexico) and the Paragon algorithm as search engine.

To determine enolase and TGase activities, purified rCaEno1 protein was passed through a Sephadex G-25 column (5.7 × 0.7 cm) and collected as a V_0_ fraction in either 15 mm phosphate buffer, pH 6.8, or 50 mm Tris-HCl, pH 7.4, respectively.

Rabbit polyclonal antibodies against rCaEno1 protein were produced as described (57), using 50 μg of purified protein in each immunization. We used rabbits from the Laboratory Animals Production and Experimentation Unit (UPEAL) at Cinvestav-IPN, following the general principles of care and use of animals approved by the Ethics Committee from UPEAL-LaNSE at Cinvestav-IPN. Rabbit polyclonal anti-rEhPCNA antibodies were obtained from the purified protein as described above, using the pRSET A-*Ehpcna* plasmid encoding the full-length open reading frame (GenBank^TM^ accession number XM_646418.1). Both antibodies were immunoadsorbed to their corresponding nitrocellulose-immobilized recombinant proteins, previously blocked with bovine serum albumin (BSA), eluted with 100 mm glycine, pH 2.3, and immediately neutralized with 1 m Tris-HCl, pH 8.0.

### Bioinformatics analysis

The primary sequence alignment of CaEno1 (GenBank^TM^ accession number AAB46358.1) and ScEno1 (GenBank^TM^ accession number AAA88712.1) was performed using the Clustal Omega program (version 1.2.4) at the European Bioinformatics Institute (http://www.ebi.ac.uk/Tools/msa/clustalo/)^5^(59) and visualized with the BoxShade program at the ExPASy Bioinformatics Resource Portal (http://www.ch.embnet.org/software/BOX_form.html).^5^ The structure of CaEno1p was obtained by homology modeling using the Modeler Program at the ModWeb Server for Protein Structure Modeling (version r189, https://modbase.compbio.ucsf.edu/modweb/),^5^ selecting the best and longest scoring model with very low slow fold assignment methods. The query protein (residues 4–440) was modeled, using as a template the corresponding region of the ScEno1p (residues 2–436), which was engineered to obtain a heterodimer composed of one active and one inactive subunit (RCSB Protein Data Bank code 2al1). Protein structures were visualized with UCSF Chimera (version 1.10.2, build 40686, https://www.cgl.ucsf.edu/chimera/).^5^ The structures were compared with the Match Maker module of Chimera, using the Needleman-Wunsch algorithm and the BLOSUM62 matrix for best-aligning pair of chains. Quality model evaluation was performed with VERIFY_3D and ERRAT programs at the Structure Analysis and Verification Server version 4 (SAVES) from the Molecular Biology Institute UCLA (http://services.mbi.ucla.edu/SAVES/).^5^ Ramachandran plots were obtained with the RAMPAGE program at the University of Cambridge (http://mordred.bioc.cam.ac.uk/∼rapper/rampage.php).^5^ The CaEno1 homology model was deposited in the Protein Model Database from CASPUR and the Biocomputing Group of the Department of Biochemical Sciences of the University of Rome “LA Sapienza” (https://bioinformatics.cineca.it/PMDB/),^5^ with the identifier PM0081054. To determine the surface electrostatic potential (EP) of CaEno1p and ScEno1p, the PDB files were converted to a PQR format using the PDB2PQR (version 2.1.1) server at the National Biomedical Computation Resource website (http://nbcr.ucsd.edu/)^5^ employing the default parameters in the program (force field, PARSE; optimization of hydrogen bonding network) and pH 6.8 (pH of enolase activity assay). The EP of molecular models was determined with Adaptive Poisson-Boltzmann Solver (APBS) software following the web link mentioned above. Finally, the EP on the protein surface was visualized with Chimera (setting values between −5 (red) to +5 (blue) kcal/mol). The BioGRID (the Biological General Repository for Interaction Datasets, https://thebiogrid.org/)^5^ and functional protein association networks with the STRING program (https://string-db.org/)^5^ (60) were used to determine interactions maps and interactors for enolase. The structural classification of enolase was performed at the CATH/Gene3D (version 4.1) website (http://www.cathdb.info/)^5^ (61). A search of domains was performed in the Protein Sequence Analysis and Classification database at the European Bioinformatics Institute (InterPro, https://www.ebi.ac.uk/interpro/).^5^

### Immunolocalization of Eno1 in C. albicans by IEM

Detection of Eno1 in *C. albicans* was achieved by electron microscopy following a procedure previously reported (58). Yeasts were washed with PBS and then fixed with a mixture of 4% paraformaldehyde and 0.05% glutaraldehyde in 0.2 m PIPES, pH 7.0, for 1 h at room temperature. After thorough washings with PBS, yeasts were blocked by incubation in 10% fetal calf serum in PBS, washed with PBS, then dehydrated, and then embedded in increasing concentrations of ethanol and LR White resin (London Resin, Polysciences, Inc.). Yeast embedded in resin were polymerized in gelatin capsules overnight, under UV light, at 4 °C. Immunogold labeling was carried out in thin sections obtained with an ultra-microtome (Reichert Jung, Austria) and then mounted on Formvar-covered nickel grids. Grids with the sections were floated on drops of the following solutions: (*a*) 0.1% skim milk and PBS-T (PBS and 0.05% Tween 20) for 30 min to diminish non-specific labeling; (*b*) rabbit antibody against Eno1 recombinant protein at a 1:20 dilution in PBS-T for 1 h at room temperature and overnight at 4 °C, and (*c*) goat anti-rabbit polyclonal antibody coupled to 10-nm gold particles for 2 h at room temperature (Zymed Laboratories Inc. and Thermo Fisher Scientific) (1:40 dilution in PBS-T). Grids were washed in every step with PBS-T and in the last step with PBS and distilled water. Sections were contrasted with 3% uranyl acetate and then examined in a transmission electron microscope (TEM, JEOL 1400x, JEOL Ltd., Japan). As negative control, sections were incubated with serum from a normal rabbit diluted in PBS-T and then with secondary antibodies coupled to gold particles. Identification of subcellular structures positive to Eno1 was performed using thin sections of yeasts that were processed to preserve the ultrastructure. Briefly, cells were fixed for 1 h in 2.5% glutaraldehyde. After thorough washings in PBS, cells were fixed for 1 h in 1% OsO_4_ at 4 °C, rinsed with PBS, gradually dehydrated in ethanol, and finally embedded in Spurr's resin. Thin sections were obtained with ultramicrotome and stained with saturated uranyl acetate dissolved in ethanol and a saturated aqueous lead citrate. Copper grids with the sections were examined in the TEM. Digital images were obtained and processed with Adobe Photoshop software.

## Author contributions

J. P. L. A., E. R. B., M. I., and S. M. designed research. J. P. L. A., E. R. B., M. I., S. M., M. L. L. B., R. M. F., and K. G. C. G. performed research. J. P. L. A., E. R. B., M. I., R. M. F., and S. M. analyzed data. J. P. L. A., E. R. B., M. I., and S. M. wrote the manuscript.

## Supplementary Material

Supporting Information

## Footnotes

4 M. Iranzo and S. Mormeneo, unpublished results.

5 Please note that the JBC is not responsible for the long-term archiving and maintenance of this site or any other third party hosted site.

## Footnotes

3 The abbreviations used are:

CWP: cell wall protein
CFW: calcofluor white
TGase: transglutaminase
EV: extracellular vesicle
PDB: Protein Data Bank
IEM: immunoelectron microscopy
MDC: monodansylcadaverine
DAPI: 4,6-diamidino-2-phenylindole
PMSF: phenylmethylsulfonyl fluoride
Ni^2+^-NTA: Ni^2+^-nitrilotriacetic acid
PEP: phosphoenolpyruvate
EP: electrostatic potential
IMAC: immobilized metal affinity chromatography
GPI: glycosylphosphatidylinositol
ASL: alkali-sensitive linkage
CW: cell wall
MMF: mixed membrane fraction
anti-rEhPCNA: anti-recombinant *E. histolytica* PCNA
TEM: transmission electron microscopy
SEM: scanning electron microscopy
SN: supernatant
WB: Western blotting.
