# Supplementary material for: The Candida albicans ENO1 gene encodes a transglutaminase involved in growth, cell division, morphogenesis, and osmotic protection
Source: J Biol Chem. 2018 Jan 31;293(12):4304–23. doi: 10.1074/jbc.M117.810440 (PMC5868267; doi:10.1074/jbc.M117.810440)
Supplement: Supporting Information [file supp_293_12_4304__index.html]

The Candida albicans ENO1 gene encodes a transglutaminase involved in growth, cell division, morphogenesis, and osmotic protection — TGase role in C. albicans biology — Supporting Information 

# The *Candida albicans ENO1* gene encodes a transglutaminase involved in growth, cell division, morphogenesis, and osmotic protection

## Supporting Information

- Supplemental Fig. S1 (.pdf, 603 KB) - Classification of proteins extracted with 2% SDS in the Gene Ontology Panther Classification System
- Supplemental Fig. S2 (.pdf, 304 KB) - Classification of proteins extracted with zymolyase in the Gene Ontology Panther Classification System
- Supplemental Fig. S3 (.pdf, 210 KB) - Classification of proteins extracted with chitinase in the Gene Ontology Panther Classification System
- Supplemental Fig. S4 (.pdf, 177 KB) - Amino acid sequence alignment of the Atg8 autophagy marker from C. albicans, S. cerevisiae and H. sapiens revealed a conserved epitope recognized by anti-Atg8 rabbit polyclonal antibody
- Supplemental Fig. S6 (.pdf, 42 KB) - Determination of cell wall TGase activity of Candida albicans digested with zymolyase and chitinase and subjected to solubilization with 8 M urea
- Supplemental Fig. S7 (.pdf, 119 KB) - Identification of the recombinant C. albicans enolase 1 by tandem mass spectrometry
- Supplemental Fig. S9 (.pdf, 89 KB) - Clustal Omega alignment of C. albicans enolase 1 and S. cerevisiae enolase 1 amino acid sequences
- Supplemental Fig. S10 (.pdf, 1.3 MB) - Structural comparison of molecular models of CaEno1 and ScEno1 polypeptides
- Supplemental Fig. S11 (.pdf, 446 KB) - Rammachandran plots of C. albicans and S. cerevisiae enolase 1 structures
- Supplemental Fig. S12 (.pdf, 376 KB) - Structural comparison of catalytic sites of CaEno1 and ScEno1 polypeptides bound to phosphoenolpyruvate/2-phophoglycerate
- Supplemental Fig. S13 (.pdf, 1.3 MB) - Alignment of C. albicans enolase 1 with TGases from Anopheles gambiae, Mus musculus, Gallus gallus, Apis mellifera, and Homo sapiens
- Supplemental Fig. S14 (.pdf, 584 KB) - Identification of a putative TGase active site in the CaEno1 structure
- Supplemental Table S1 (.pdf, 26 KB) - Comparison between radioactive putrescine and lysine used as substrates for the determination of TGase activity from guinea pig liver
- Supplemental Table S2 (.xls, 728 KB) - Proteins identified in the SDS-PAGE gel fragment containing the 14C-Lysine labeled proteins that were extracted with 2% SDS
- Supplemental Table S3 (.xls, 74 KB) - Proteins identified in the SDS-PAGE gel fragment containing the 14C-Lysine labeled proteins that were extracted with zymolyase
- Supplemental Table S4 (.xls, 75 KB) - Proteins identified in the SDS-PAGE gel fragment containing the 14C-Lysine labeled proteins that were extracted with chitinase
- Supplemental Table S5 (.xls, 70 KB) - PROTEINS IDENTIFIED IN THE FLUORESCENT BANDS INDICATED IN FIGURE 6, C AND D
- Supplemental Table S6 (.xls, 590 KB) - PEPTIDES IDENTIFIED IN THE FLUORESCENT BANDS INDICATED IN FIGURE 6, C AND D
- Supplemental methods (.pdf, 99 KB) - Methods used in mass spectrometry analysis of 14C-lysine labeled proteins and immunofluorescence
- Supporting Information (.jpg, 183 KB)
- Supporting Information (.jpg, 194 KB)
