# Supplementary material for: The Candida albicans ENO1 gene encodes a transglutaminase involved in growth, cell division, morphogenesis, and osmotic protection
Source: J Biol Chem. 2018 Jan 31;293(12):4304–23. doi: 10.1074/jbc.M117.810440 (PMC5868267; doi:10.1074/jbc.M117.810440)
Supplement: Supporting Information [file 10.1074_M117.810440_jbc.M117.810440-2.pdf]

A

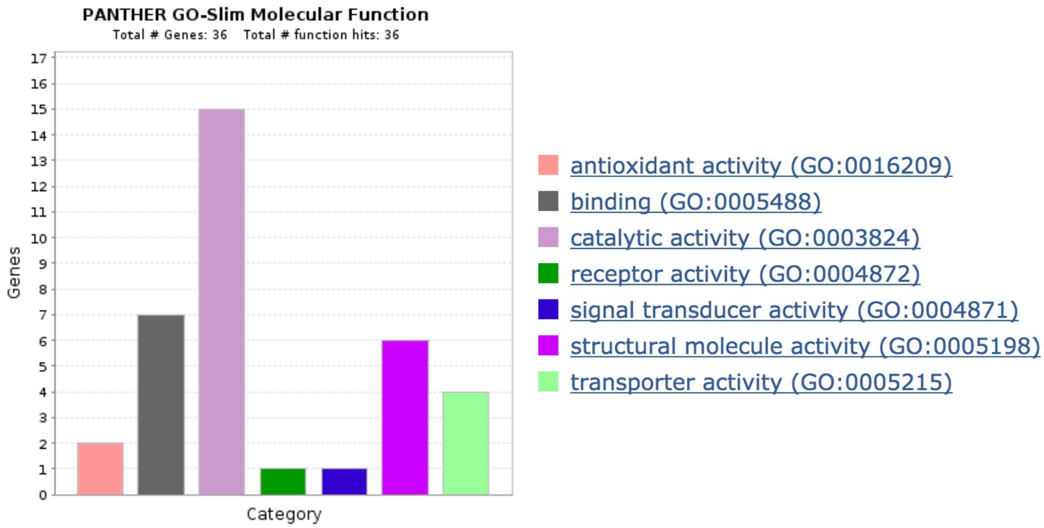

B

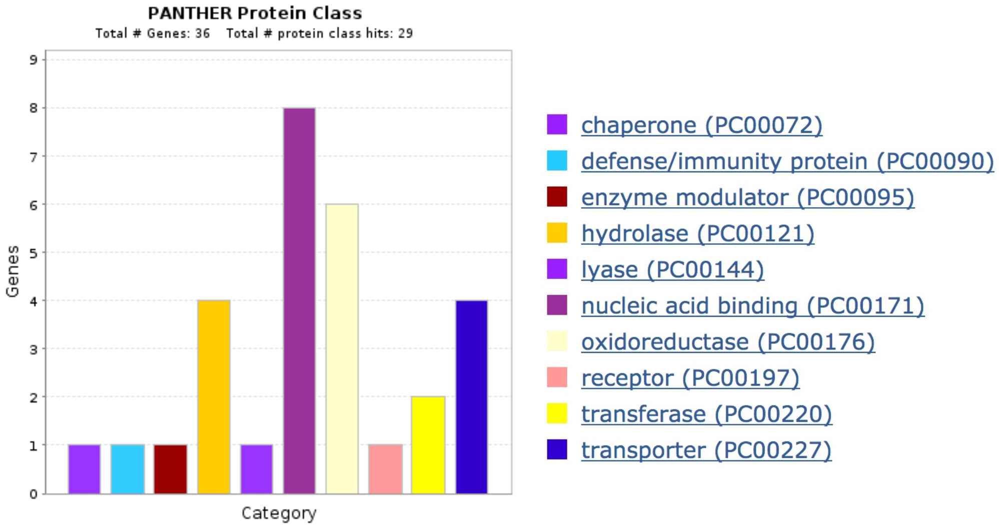

Figure S2. Classification of the proteins extracted with zymolyase in the Gene Ontology Panther Classification System. The 37 proteins were categorized according to their Molecular Function (**A**) and Protein Class (**B**).
