# Supplementary material for: The Candida albicans ENO1 gene encodes a transglutaminase involved in growth, cell division, morphogenesis, and osmotic protection
Source: J Biol Chem. 2018 Jan 31;293(12):4304–23. doi: 10.1074/jbc.M117.810440 (PMC5868267; doi:10.1074/jbc.M117.810440)
Supplement: Supporting Information [file 10.1074_M117.810440_jbc.M117.810440-4.pdf]

```
HsLC3     1  MPSDRPFKQRRSFADRCKEVQQIRDQHPSKIPVIIERYKGEKQLPVLDKTKFLVPDHVNM
CaAtg8    1  --MRSQFKDEHPFEKRQAEAARIAQRFKDRVPVICEKVE-NSDIPEIDKRKYLVPVDLTV
ScAtg8    1  --MKSTFKSEYPFEKRKAESERIADRFKNRIPVICEKAE-KSDIPEIDKRKYLVPADLTV

HsLC3    61  SELVKIIRRRLQLNPTQAFFLLVNQHSMVSVSTPIADIYEQEKDEDGFLYMVYASQETFG
CaAtg8   58  GQFVYVIRKRIKLPSEKAIFIFVNDIL-PPTAALISTIYEEHKDEDGFLYVLYSGENTFG
ScAtg8   58  GQFVYVIRKRIMLPPEKAIFIFVNDTL-PPTAALMSAIYQEHKDKDGFLYVTYSGENTFG

HsLC3   121  F------------------
CaAtg8  117  EKLAIDISSLDFSDIPDYV
ScAtg8  117  R------------------
```

**Figure S4.** Amino acid sequence alignment of the Atg8 autophagy marker from *C. albicans*, *S. cerevisiae* and *H. sapiens* revealed a conserved epitope recognized by anti-Atg8 rabbit polyclonal antibody (rectangle).
