# Supplementary material for: The Candida albicans ENO1 gene encodes a transglutaminase involved in growth, cell division, morphogenesis, and osmotic protection
Source: J Biol Chem. 2018 Jan 31;293(12):4304–23. doi: 10.1074/jbc.M117.810440 (PMC5868267; doi:10.1074/jbc.M117.810440)
Supplement: Supporting Information [file 10.1074_M117.810440_jbc.M117.810440-5.pdf]

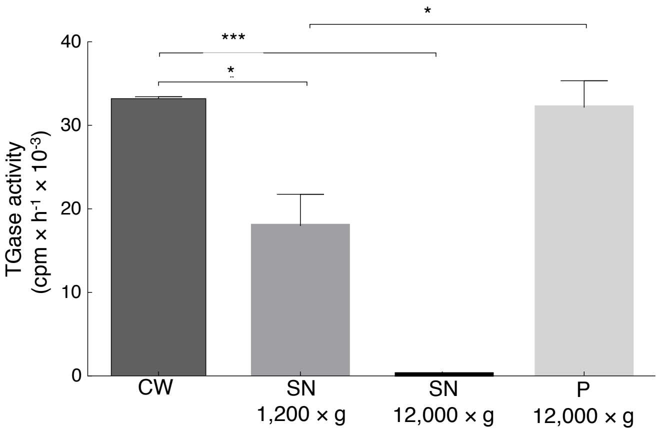

Figure S6. Determination of cell wall TGase activity of *Candida albicans* digested with zymolyase and chitinase and subjected to solubilization with 8 M urea. Cell walls of *Candida albicans* were digested with zymolyase and chitinase, denatured with 8 M urea, renatured by dialysis, centrifuged to obtain different fractions, and determined TGase activity in each of them as described in Experimental Procedures. CW, cell walls; SN, supernatant; P, pellet.
