# Supplementary material for: The Candida albicans ENO1 gene encodes a transglutaminase involved in growth, cell division, morphogenesis, and osmotic protection
Source: J Biol Chem. 2018 Jan 31;293(12):4304–23. doi: 10.1074/jbc.M117.810440 (PMC5868267; doi:10.1074/jbc.M117.810440)
Supplement: Supporting Information [file 10.1074_M117.810440_jbc.M117.810440-6.pdf]

A

Peptides identified by tandem mass spectrometry in the purified rCaEno1:

SIVPSGASTGVHEALELR
$[MW + H]^+$ = 1822.8105

LGANAILGVSLAAANAAAAAQGIPLYK
$[MW + H]^+$ = 2509.4063

VNQIGTLTESIQAANDSYAAGWGVMVSHR
$[MW + H]^+$ = 3075.2451

SGETEDTFIADLSVGLR
$[MW + H]^+$ = 1809.7429

>CaEno1
MSYATKIHARYVYDSRGNPTVEVDFTTDKGLFR**SIVPSGASTGVHEALELR**DGDKSKW
LGKGVLKAVANVNDIIAPALIKAKIDVVDQAKIDEFLLSLDGTPNKSK**LGANAILGVSLAA**
ANAAAAAQGIPLYKHIANISNAKKGKFVLPVPFQNVLNGGSHAGGALAFQEFMIAPTG
VSTFSEALRIGSEVYHNLKSLTKKKYGQSAGNVGDEGGVAPDIKTPKEALDLIMDAIDKA
GYKGKVGIAMDVASSEFYKDGKYDLDFKNPESDPSKWLSGPQLADLYEQLISEYPIVSI
EDPFAEDDWDAWVHFFERVGDKIQIVGDDLTVTNPTRIKTAIEKKAANALLLK**VNQIGTL**
TESIQAANDSYAAGWGVMVSHRSGETEDTFIADLSVGLRSGQIKTGAPARSERLAKLN
QILRIEEELGSEAIYAGKDFQKASQL

B

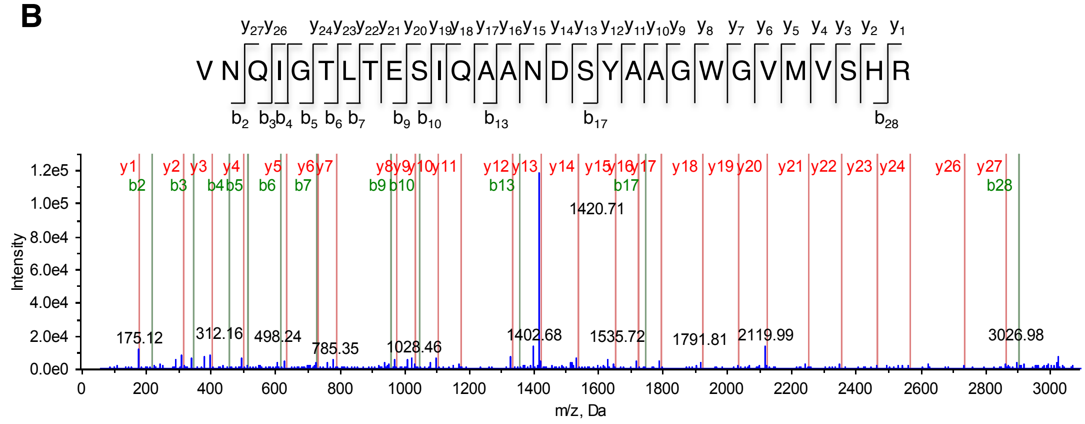

Figure S7. Identification of the recombinant *C. albicans* enolase 1 (rCaEno1) by tandem mass spectrometry. *A,* amino acid sequences are shown for the peptides identified (bold letters) by mass spectrometry with more than 95% in confidence, covering 20.68% of the total CaEno1 amino acid sequence. The m/z ratios for the corresponding precursor ions are shown. *B*, mass spectrum of the QIGTLTESIQAANDSYAAGWGVMVSHR peptide is shown as an example.
