# Supplementary material for: The Candida albicans ENO1 gene encodes a transglutaminase involved in growth, cell division, morphogenesis, and osmotic protection
Source: J Biol Chem. 2018 Jan 31;293(12):4304–23. doi: 10.1074/jbc.M117.810440 (PMC5868267; doi:10.1074/jbc.M117.810440)
Supplement: Supporting Information [file 10.1074_M117.810440_jbc.M117.810440-7.pdf]

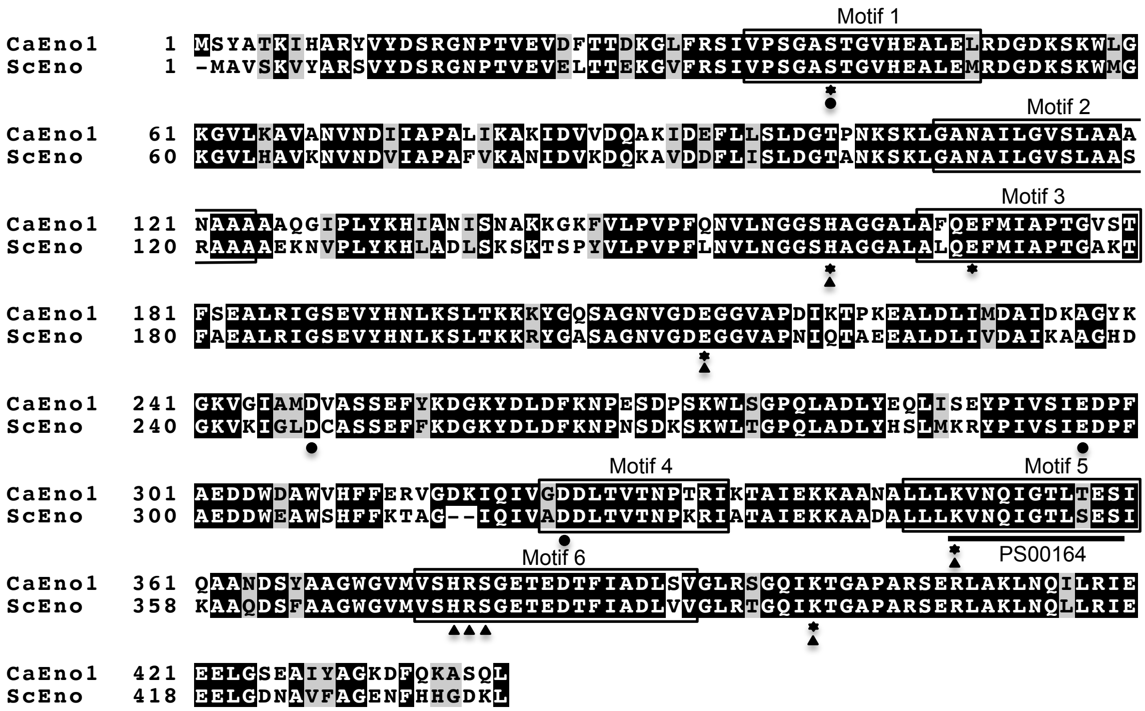

Figure S9. Clustal Omega alignment of *C. albicans* enolase 1 and *S. cerevisiae* enolase 1 amino acid sequences. Identical residues are highlighted in black and conserved residues are highlighted in gray. Residues in rectangles correspond to Motifs 1-6 found in enolase family (PRINTS code: PR00148). Underlined residues correspond to PS000164 PROSITE conserved motif found in enolase. According to cd03313 conserved protein domain family: circles, metal-binding residues; triangles, substrate-binding residues; asterisks, catalytic-site residues.
