# Supplementary material for: The Candida albicans ENO1 gene encodes a transglutaminase involved in growth, cell division, morphogenesis, and osmotic protection
Source: J Biol Chem. 2018 Jan 31;293(12):4304–23. doi: 10.1074/jbc.M117.810440 (PMC5868267; doi:10.1074/jbc.M117.810440)
Supplement: Supporting Information [file 10.1074_M117.810440_jbc.M117.810440-8.pdf]

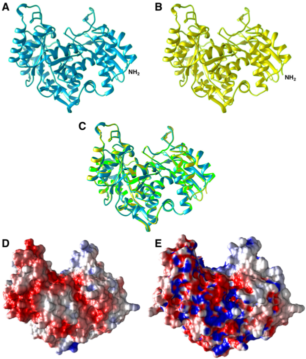

Figure S10. Structural comparison of molecular models of CaEno1 and ScEno1 polypeptides. The structure of CaEno1 was theoretically determined by homology modeling with the Modeller program using the structure of ScEno1 as a template corresponding to a heterodimer composed of one active and one inactive subunit (RCSB PDB ID 2al1) and visualized with the Chimera program. CaEno1 structure was only compared to active subunits (*A, B*) Cyan and yellow ribbon models for CaEno1 and ScEno1 proteins, respectively. (*C*) Superimposed ribbon structure models for CaEno1 and ScEno1. (*D*, *E*) Electrostatic potential on surface of CaEno1 and ScEno1 molecules calculated at pH 6.8, respectively.
