# Supplementary material for: The Candida albicans ENO1 gene encodes a transglutaminase involved in growth, cell division, morphogenesis, and osmotic protection
Source: J Biol Chem. 2018 Jan 31;293(12):4304–23. doi: 10.1074/jbc.M117.810440 (PMC5868267; doi:10.1074/jbc.M117.810440)
Supplement: Supporting Information [file 10.1074_M117.810440_jbc.M117.810440-9.pdf]

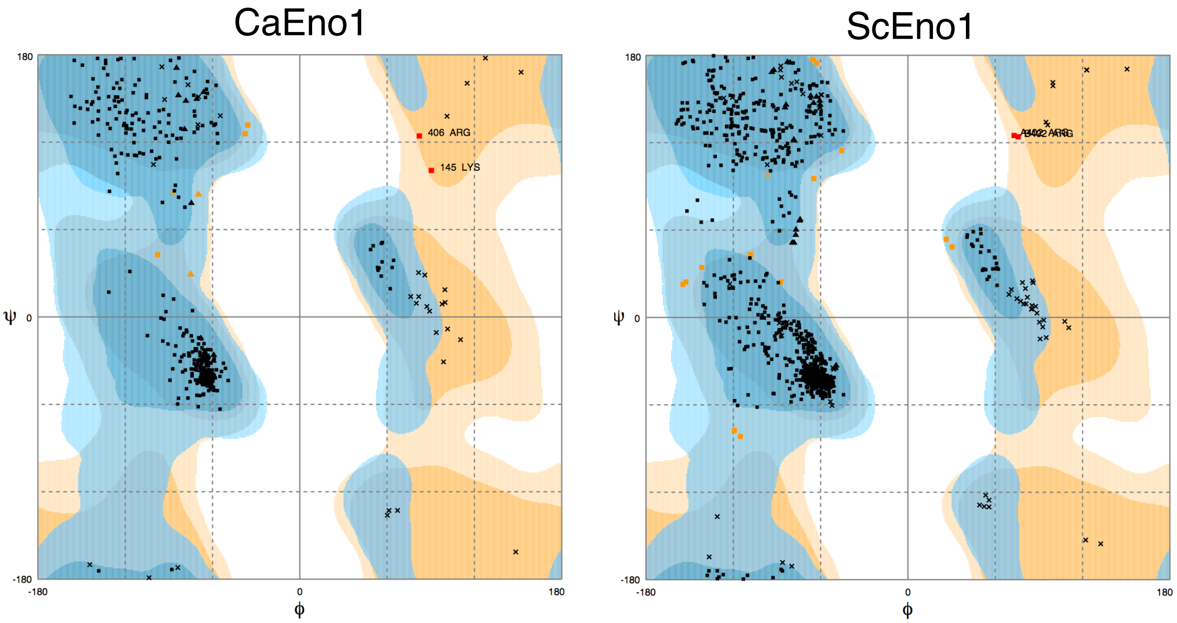

Figure S11. Rammachandran plots of *C. albicans* and *S. cerevisiae* enolase 1 structures. The CaEno1 protein structure has 99.6% of all residues in favored (98.2%) and allowed (1.4%) regions. The ScEno1 (PDB ID 2al1) protein structure has 99.7% of all modeled residues in favored (98.1%) and allowed (1.6%) regions.
