# Supplementary material for: The Candida albicans ENO1 gene encodes a transglutaminase involved in growth, cell division, morphogenesis, and osmotic protection
Source: J Biol Chem. 2018 Jan 31;293(12):4304–23. doi: 10.1074/jbc.M117.810440 (PMC5868267; doi:10.1074/jbc.M117.810440)
Supplement: Supporting Information [file 10.1074_M117.810440_jbc.M117.810440-10.pdf]

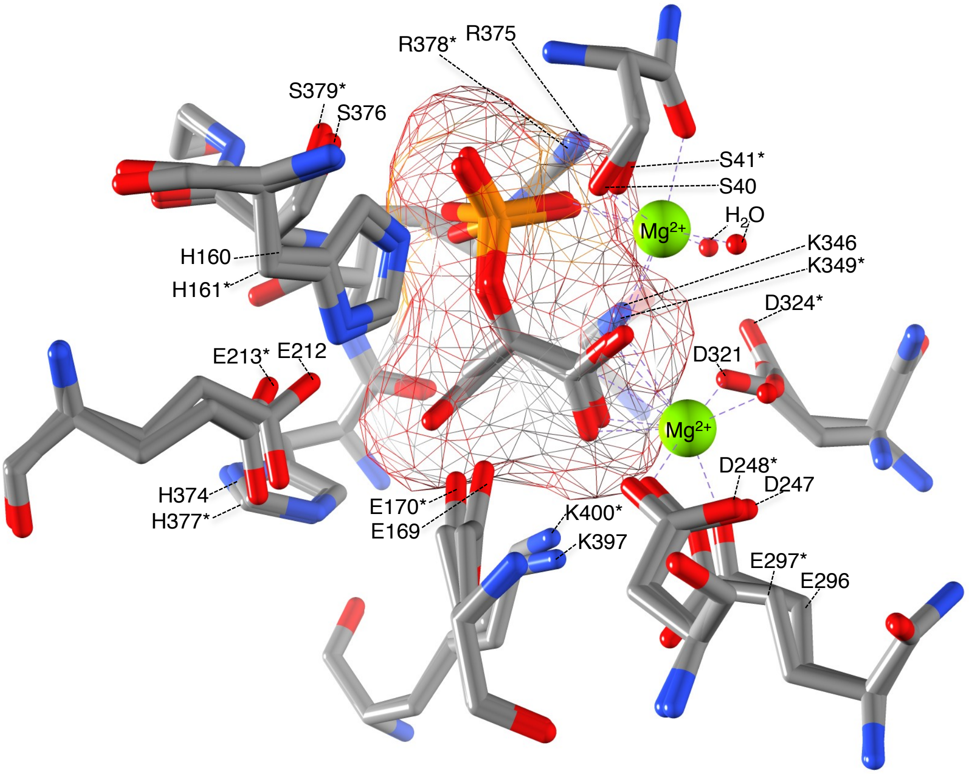

Figure S12. Structural comparison of catalytic sites of CaEno1 and ScEno1 polypeptides bound to phosphoenolpyruvate/2-phophoglycerate. Surface of substrate/product is represented by a mesh. Asterisks, residues corresponding to *C. albicans* enolase 1.
