# Supplementary material for: The Candida albicans ENO1 gene encodes a transglutaminase involved in growth, cell division, morphogenesis, and osmotic protection
Source: J Biol Chem. 2018 Jan 31;293(12):4304–23. doi: 10.1074/jbc.M117.810440 (PMC5868267; doi:10.1074/jbc.M117.810440)
Supplement: Supporting Information [file 10.1074_M117.810440_jbc.M117.810440-11.pdf]

```
CaEno1        1 ------------------------------------------------------------
AgTGase       1 ------------------------------------------------------------
MmTGase       1 ------------------------------------------------------------
GgTGase       1 ------------------------------------------------------------
AmTGase       1 ------------------------------------------MFY-RFYD----------
HsTGase       1 MMDGPRSDVGRWGGNPLQPPTTPSPEPEPEPDGRSRRGGGRSFWARCCGCCSCRNAADDD
HsFactorXII   1 ------------------------------------------------------------

CaEno1        1 ------------------------------------------------------------
AgTGase       1 ---MSTTSTNYRIWPARK----------------------------VVTNSAIKDVLKIE
MmTGase       1 -----------------------------------------------MAEELLL-----E
GgTGase       1 ------------------------------------MG--GPGPDGTMAEELVL-----E
AmTGase       8 ---------RYRIFNDRRFRP---------------------FPFAFYWEDFTVPVLTIL
HsTGase      61 WGPEPSDSRGRGSSSGTRR---PGSRGSDSRRPVSRGSGVNAAGDGTIREGMLV-VNGVD
HsFactorXII   1 --SE----TSRTAFGGRRAVPPNNSNAAEDDLPTVELQGV--VPRGVNLQEFLN-VTSVH

CaEno1        1 ----------------------------------MSYATKIHARYVYDSRGNPTVEVDFT
AgTGase      30 SINLCFDENGKTFHTSHYEAMTKPRPTKRPRQLIVRRGAPFEVRLLT-NRRFDP-----T
MmTGase       9 RCDLEIQANGRDHHTADL----------CQEKLLVRRGQRFRLTLYFEGRGYQA-----S
GgTGase      18 TCDLQCERNGREHRTEEM----------GSQQLVVRRGQPFTITLNFAGRGYEE-----G
AmTGase      38 HVDPCIGENGAKHRTSRYEAMSRNG---DGSRLVVRRGQEFYLHLAL-SRDYDP-----D
HsTGase     117 LLSSRSDQNRREHHTDEY----------EYDELIVRRGQPFHMLLLL-SRTYES-----S
HsFactorXII  52 LFKERWDTNKVDHHTDKY----------ENNKLIVRRGQSFYVQIDF-SRPYDP-----R

CaEno1       27 TDKGLFRSIVPSG-A-----STGVHEAL--ELR---------DGDKSKWLGKGVLK----
AgTGase      84 VDTMVLVVSIVSYGSEKPCFGNGTETYVLVSVATDGTAPAEPDELAEDWRAVVVDSKDKE
MmTGase      54 VDSLTFGAVT--G--PDPSEEAGTKARF--SLSDN------V--EEGSWSASVLDQQDN-
GgTGase      63 VDKLAFDVET--G--PCPVETSGTRSHF--TLTDC------P--EEGTWSAVLQQQDGA-
AmTGase      89 TDGISLVFTV--DGAERPQYGHGTLVAT--PVLYP------GQRSEASWQAYIDSLHSN-
HsTGase     161 -DRITLELLI--G--NNPEVGKGTHVII--PVG-K------GG--SGGWKAQVVKASGQ-
HsFactorXII  96 RDLFRVEYVI--G--RYPQENKGTYIPV--PIVSE------LQ--SGKWGAKIVMREDR-

CaEno1       66 -----------------------------------AVANVN----DIIAPALIKAKIDVV
AgTGase     144 KGKVELTLQIMIPSYTPVWRWSIQFHTRLDTTDAKSMTEIKEPLFVLFNPWCKNDAVYME
MmTGase      99 ----VLSLQLCTPANAPIGLYRLSLEASTGYQ---GSSFVLGHFILLYNAWCPADDVYLD
GgTGase     108 ----TLCVSLCSPSIARVGRYRLTLEASTGYQ---GSSFHLGDFVLLFNAWHPEDAVYLK
AmTGase     138 ----FLRLKIVPAPDAIIGKWRMDIDTKNKSTDGAVSFTMKHPFYLVFNPWCKEDAVYME
HsTGase     204 ----NLNLRVHTSPNAIIGKFQFTVRTQSDAGEFQLPFDPRNEIYILFNPWCPEDIVYVD
HsFactorXII 141 ----SVRLSIQSSPKCIVGKFRMYVAVWTPYGVLRTSRNPETDTYILFNPWCEDDAVYLD

CaEno1       87 DQAKIDEFLLSLDGTPNKSKLG------------ANAILGVSL----------AAANAAA
AgTGase     204 DEAWRSEYILDDSTAICKPASKGLHMSSWFLGQFEANVLDSALYIVEQV------ANVKA
MmTGase     152 SEEERREYVLTQQGFIYQGSVKFIKSVPWNFGQFQDGILDTCLMLLDMNPKFLKNRSRDC
GgTGase     161 EEDERREYVLSQQGLIYMGSRDYITSTPWNFGQFEDEILAICLEMLDINPKFLRDQNLDC
AmTGase     194 GEDERQEYVMAEDGLIWRGSYNRPRRTVWKYSQFERDILDCALHLMIEV------GKVRV
HsTGase     260 HEDWRQEYVLNESGRIYYGTEAQIGERTWNYGQFDHGVLDACLYILDR-------RGMPY
HsFactorXII 197 NEKEREEYVLNDIGVIFYGEVNDIKTRSWSYGQFEDGILDTCLYVMDR-------AQMDL

CaEno1      125 AAQGIPLYK--HIANISNAKK-GKFVLPVPFQNVLNGGSHA----GGALAFQEFM-----
AgTGase     258 PISGNPVLVIRALSGALNTAD-GTGVLQGNWSNNYEGGTAPTSWTGSAKILQEFYDTG-E
MmTGase     212 SRRSSPIYVGRVVSDMVNCND-DQGVLLGRWDNNYGDGISPMAWIGSVDILRRWKEHGCQ
GgTGase     221 SRRNDPVYIGRVVSAMVNCNDEDHGVLLGRWDNHYEDGMSPMAWIGSVDILKRWRRLGCQ
AmTGase     248 SARHDPVIISRVLSAAVNSPD-DNGALMGNWSNDFGGGTPPTKWLGSQKILQQYYKTR-K
HsTGase     313 GGRGDPVNVSRVISAMVNSLD-DNGVLIGNWSGDYSRGTNPSAWVGSVEILLSYLRTG-Y
HsFactorXII 250 SGRGNPIKVSRVGSAMVNAKD-DEGVLVGSWDNIYAYGVPPSAWTGSVDILLEYRSSE-N

CaEno1      173 --------IAPTGVSTFSEALRIGSEVYHNLKSLTKKKYGQSAGNVGDEGGVAPDIKTPK
AgTGase     316 RVKYGQCWVYAGLLTSVCRAIGIPSRIITNFESAGDHDASLS------------------
MmTGase     271 QVKYGQCWVFAAVACTVLRCLGIPTRVVTNYNSAHDQNSNLL------------------
GgTGase     281 PVKYGQCWVFAAVACTVMRCLGVPSRVVTNYNSAHDTNGNLV------------------
AmTGase     306 PVKYGQCWVFSGVLATICRALGLPCRVVTNYSSAHDTQSSLT------------------
HsTGase     371 SVPYGQCWVFAGVTTTVLRCLGLATRTVTNFNSAHDTDTSLT------------------
HsFactorXII 308 PVRYGQCWVFAGVFNTFLRCLGIPARIVTNYFSAHDNDANLQ------------------
```

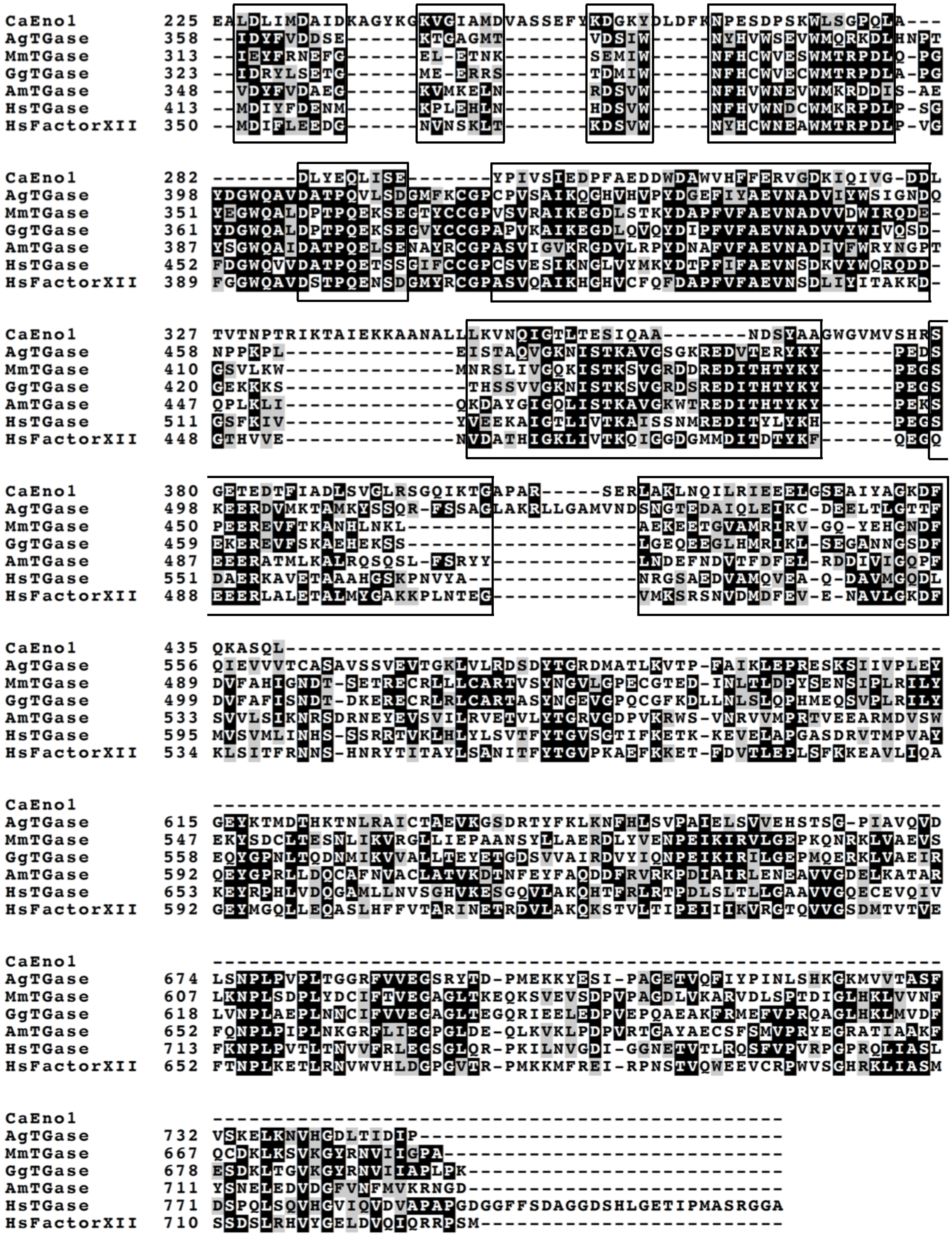

Figure S13. Clustal Omega alignment of *C. albicans* enolase 1 with TGases from *Anopheles gambiae* (Ag), *Mus musculus* (Mm), *Gallus gallus* (Gg), *Apis mellifera* (Am) and *Homo sapiens* (Hs).
