# Supplementary material for: The Candida albicans ENO1 gene encodes a transglutaminase involved in growth, cell division, morphogenesis, and osmotic protection
Source: J Biol Chem. 2018 Jan 31;293(12):4304–23. doi: 10.1074/jbc.M117.810440 (PMC5868267; doi:10.1074/jbc.M117.810440)
Supplement: Supporting Information [file 10.1074_M117.810440_jbc.M117.810440-12.pdf]

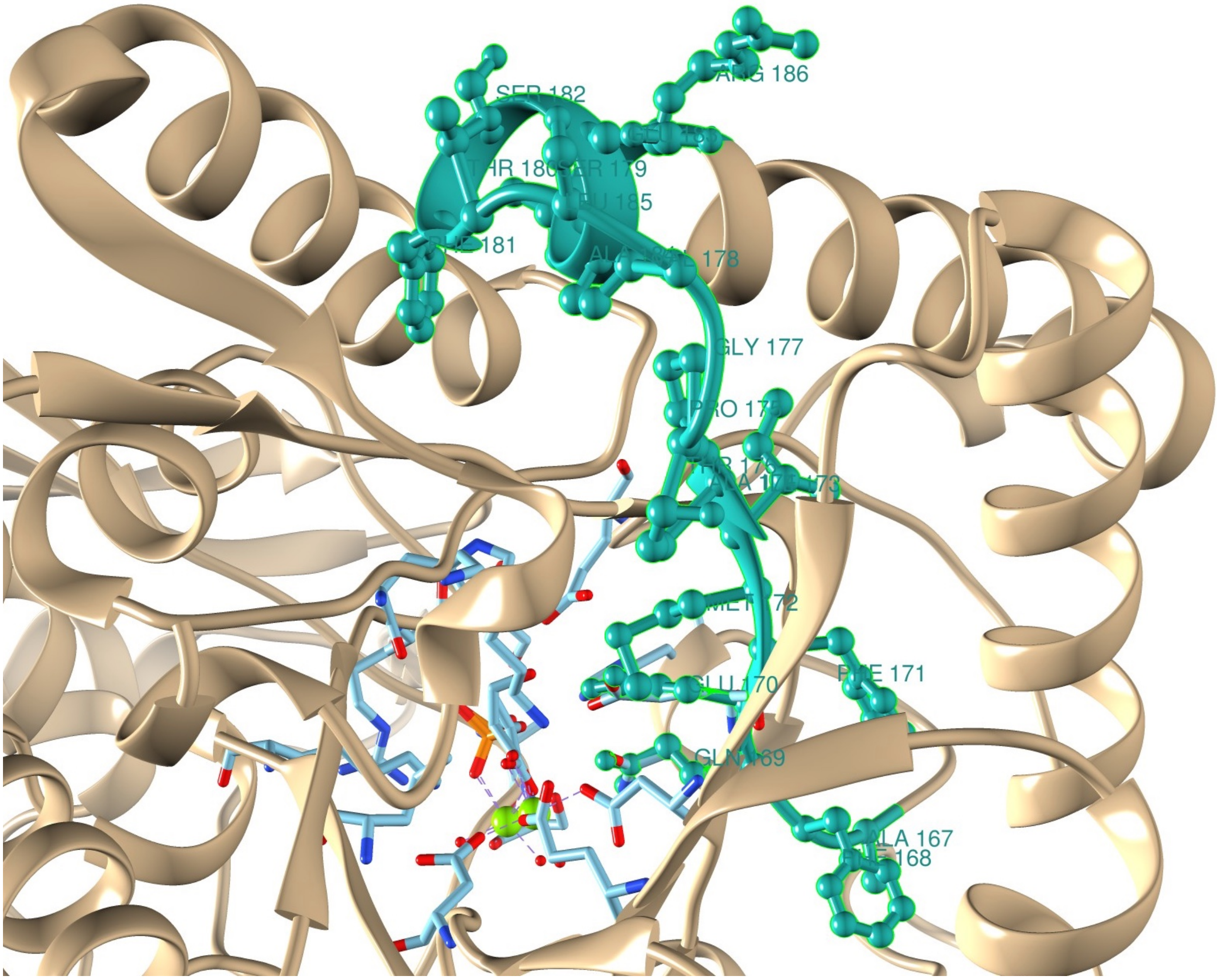

Figure S14. Identification of a putative TGase active site in the CaEno1 structure (green), showing the peptide AFQEFMIAPTGVSTFSEALR (residues 167-186) that corresponds to the PROSITE PS00547 TGase active ([GT]-Q-[CA]-W-V-x-[SA]-[GAS]-[IVT]-x(2)-T-x-[LMSC]-R-[CSAG]-[LV]-G) (http://prosite.expasy.org/PS0054).
