# Supplementary material for: The Candida albicans ENO1 gene encodes a transglutaminase involved in growth, cell division, morphogenesis, and osmotic protection
Source: J Biol Chem. 2018 Jan 31;293(12):4304–23. doi: 10.1074/jbc.M117.810440 (PMC5868267; doi:10.1074/jbc.M117.810440)
Supplement: Supporting Information [file 10.1074_M117.810440_jbc.M117.810440-13.pdf]

TGase (units)	Putrescine	Putrescine plus cystamine	Lysine	Lysine plus cystamine
	(cpm)			
0.025	6,395	200	4,074	156
0.050	13,160	128	7,615	174

Table S1. Comparison between radioactive putrescine and lysine used as substrates for the determination of TGase activity from guinea pig liver. TGase activity was determined by measuring the amounts of radioactive precursors incorporated into N,N'-dimethyl casein precipitable by TCA. Results are the average of six determinations from three experiments.
