# Supplementary material for: The Candida albicans ENO1 gene encodes a transglutaminase involved in growth, cell division, morphogenesis, and osmotic protection
Source: J Biol Chem. 2018 Jan 31;293(12):4304–23. doi: 10.1074/jbc.M117.810440 (PMC5868267; doi:10.1074/jbc.M117.810440)
Supplement: Supporting Information [file 10.1074_M117.810440_jbc.M117.810440-19.pdf]

Supplemental information

Experimental procedures

Mass Spectrometry Analysis of ^{14}C-lysine labeled proteins

We performed the sequential extraction of cell wall proteins with SDS, zymolyase and chitinase as described in Experimental procedures of the main text. The region labeled and extracted with 2% SDS was cut in five bands 2 mm width, while the each labeled region obtained with zymolyase or chitinase was cut in three sections 2 mm width. Proteins in each segment were enzymatically digested with trypsin according to the modified protocol of Shevchenko et al. (1). Resulting tryptic peptides from each gel fragment were concentrated to 20 µL. Four µL of each sample were loaded into a Symmetry C18 Trap V/M precolumn (180 µm × 20 mm, 100 Å pore size, 5 µm particle size, Waters, Milford, MA) and desalted using as a mobile phase A, 0.1% formic acid (FA) in H_2O and mobile phase B, 0.1% FA in acetonitrile (ACN) under the following isocratic gradient: 99.9% mobile phase A and 0.1% of mobile phase B at a flow rate of 5 µL/min during 3 min. Then, peptides were loaded and separated on a HSS T3 C18 Column (75 µm × 150 mm, 100 Å pore size, 1.8 µm particle size Waters, Milford, MA) using an UPLC ACQUITY M-Class (Waters, Milford, MA) at the Genomics, Proteomics and Metabolomisc Unit at the National Laboratory of Experimental Services (LaNSE) at Cinvestav-IPN, using the same mobile phases under the following gradient: 0 min 7% B (93% A), 30.37 min 40% B (60% A), 32.03-35.34 min 85% B (15% A), 37-47 min 7% B (93% A) at a flow of 400 nL/min at 45 °C. Spectra were acquired in a mass spectrometer with electrospray ionization (ESI) and ion mobility separation (IMS) Synapt G2-Si (Waters, Milford, MA) using Data-Independent Acquisition (DIA) strategy by HDMS^E technology (Waters, Milford, MA). The tune page for the ionization source was set with the following parameters: 2.75 kV in the sampler capilar, 30 V in the sampling cone, 30 V in the source offset, 70 °C for the source temperature, 0.5 Bar for the nanoflow gas and 150 L/h for the purge gas flow. Two chromatograms were acquired (low and high-energy chromatograms) in positive mode in a range of m/z 50-2000 with a velocity of 0.5 scans/s. No collision energy was applied to obtain the low-energy chromatogram, while for the high-energy chromatograms, the precursor ions were fragmented in the transfer cell using a collision energy ramp of 19-55 V. Generated .raw files were deconvoluted and compared using ProteinLynx Global SERVER (PLGS) v 3.0.3 software (2) (Waters, Milford, MA) against a reversed *Candida albicans* database (downloaded from Uniprot, 6035 protein sequences, last modification on march 9 of 2017). Workflow parameters were trypsin as a cut enzyme, one missed cleavages allowed; carbamidomethyl (C) as a fixed modification and acetyl (K), acetyl (N-term), amidation (N-term), deamidation (N, Q), oxidation (M), Phosphoryl (S, T, Y) as variable modifications. Automatic peptide and fragment tolerance, minimum fragment ion matches per peptide: 2; minimum fragment ion matches per protein: 5; minimum peptide matches per protein: 1; false discovery rate of 4%. All identifications had ≥ 95% of reliability and Synapt G2-Si was calibrated with [Glu^1]-Fibrinopeptide, $[M+2H]^{2+}$= 785.84261 at ≤ 1 ppm.

Fluorescence microscopy

Yeast cells grown in different concentrations of cystamine (0, 50, 100 and 200 mM) for 6 h were processed to fluorescence microscopy as described (4). Anti-LC3A/B antibodies were used at 1:250 dilution, while FITC-labeled secondary antibodies at 1:100 dilution. Then, cells were observed

through an Olympus BX40 fluorescence microscope. Image processing and contrast enhancement were performed with ImageJ software.

REFERENCES

1. Shevchenko, A., Tomas, H., Havlis, J., Olsen, J.V., and Mann M. (2006). In-gel digestion for mass spectrometric characterization of proteins and proteomes. *Nature protocols* **1**, 2856-2860
2. Li, G.Z., Vissers, J.P., Silva, J.C., Golick, D., Gorenstein, M.V., and Geromanos, S. J. (2009) Database searching and accounting of multiplexed precursor and product ion spectra from the data independent analysis of simple and complex peptide mixtures. *Proteomics* **9**, 1696-1719
3. Zinzalla, V., Graziola, M., Mastriani, A., Vanoni, M., and Alberghina, L. (2007). Rapamycin-mediated G1 arrest involves regulation of the Cdk inhibitor Sic1 in *Saccharomyces cerevisiae*. *Mol Microbiol*. **63**, 1482-1494
4. Pringle, J. R., Preston, R. A., Adams, A. E. M., Stearns, T., Drubin, D. G., Haarer, B. K., and Jones, E. W. (1989) Fluorescence microscopy for yeast. *Methods Cell Biol*. **31**, 357-435
