# Supplementary figures and images for: The Candida albicans ENO1 gene encodes a transglutaminase involved in growth, cell division, morphogenesis, and osmotic protection
Source: J Biol Chem. 2018 Jan 31;293(12):4304–23. doi: 10.1074/jbc.M117.810440 (PMC5868267; doi:10.1074/jbc.M117.810440)

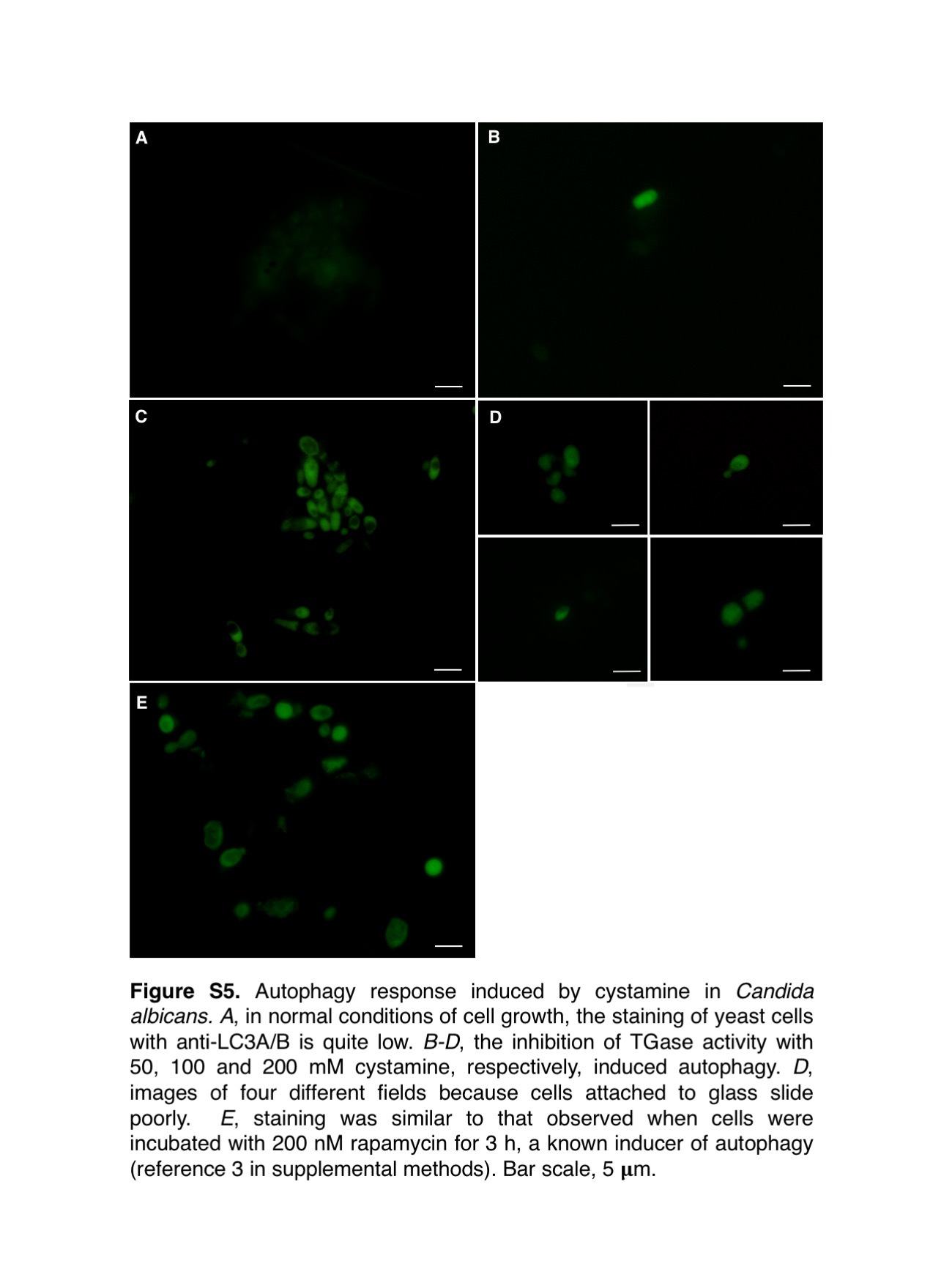

Supplement: Supporting Information [file 10.1074_M117.810440_jbc.M117.810440-20.jpg]

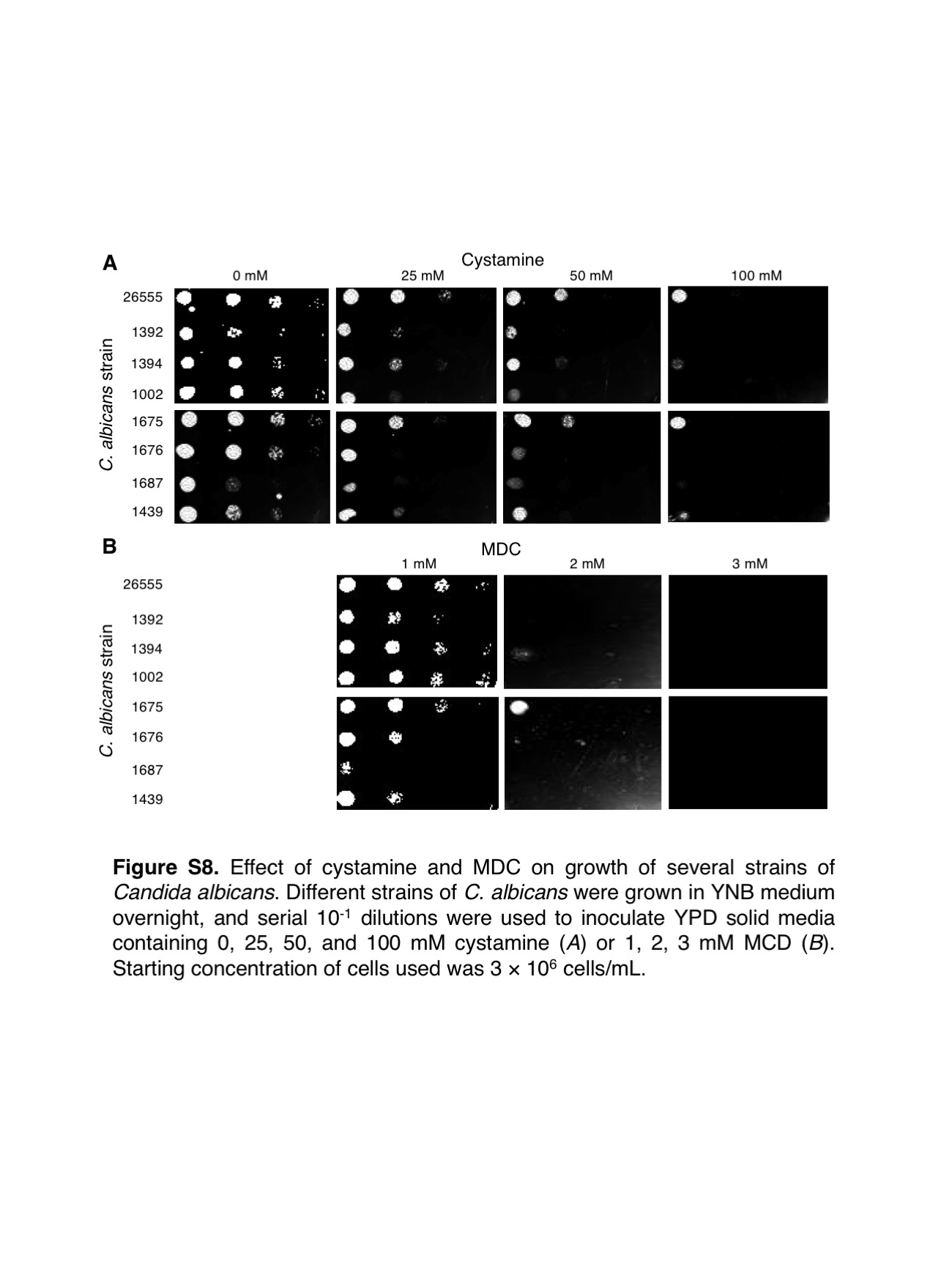

Supplement: Supporting Information [file 10.1074_M117.810440_jbc.M117.810440-21.jpg]
